# siRNA treatment targeting integrin α11 overexpressed via EZH2-driven axis inhibits drug-resistant breast cancer progression

**DOI:** 10.1186/s13058-024-01827-4

**Published:** 2024-04-25

**Authors:** Prakash Chaudhary, Kiran Yadav, Ho Jin Lee, Keon Wook Kang, Jongseo Mo, Jung-Ae Kim

**Affiliations:** 1 College of Pharmacy, Yeungnam University, Gyeongsan, 38541 Republic of Korea; 2 College of Pharmacy and Research Institute of Pharmaceutical Sciences, Seoul National University, Seoul, 08826 Republic of Korea

**Keywords:** Integrin α11, EZH2, GLI-1, HIF1α, Drug resistant breast cancer, Cancer stem cells, Epithelial-mesenchymal transition

## Abstract

**Background:**

Breast cancer, the most prevalent cancer in women worldwide, faces treatment challenges due to drug resistance, posing a serious threat to patient survival. The present study aimed to identify the key molecules that drive drug resistance and aggressiveness in breast cancer cells and validate them as therapeutic targets.

**Methods:**

Transcriptome microarray and analysis using PANTHER pathway and StemChecker were performed to identify the most significantly expressed genes in tamoxifen-resistant and adriamycin-resistant MCF-7 breast cancer cells. Clinical relevance of the key genes was determined using Kaplan-Meier survival analyses on The Cancer Genome Atlas dataset of breast cancer patients. Gene overexpression/knockdown, spheroid formation, flow cytometric analysis, chromatin immunoprecipitation, immunocytochemistry, wound healing/transwell migration assays, and cancer stem cell transcription factor activation profiling array were used to elucidate the regulatory mechanism of integrin α11 expression. Tumour-bearing xenograft models were used to demonstrate integrin α11 is a potential therapeutic target.

**Results:**

Integrin α11 was consistently upregulated in drug-resistant breast cancer cells, and its silencing inhibited cancer stem cells (CSCs) and epithelial-mesenchymal transition (EMT) while restoring sensitivity to anticancer drugs. HIF1α, GLI-1, and EZH2 contributed the most to the regulation of integrin α11 and EZH2 expression, with EZH2 being more necessary for EZH2 autoinduction than HIF1α and GLI-1. Additionally, unlike HIF1α or EZH2, GLI-1 was the sole transcription factor activated by integrin-linked focal adhesion kinase, indicating GLI-1 as a key driver of the EZH2-integrin α11 axis operating for cancer stem cell survival and EMT. Kaplan-Meier survival analysis using The Cancer Genome Atlas (TCGA) dataset also revealed both EZH2 and integrin α11 could be strong prognostic factors of relapse-free and overall survival in breast cancer patients. However, the superior efficacy of integrin α11 siRNA therapy over EZH2 siRNA treatment was demonstrated by enhanced inhibition of tumour growth and prolonged survival in murine models bearing tumours.

**Conclusion:**

Our findings elucidate that integrin α11 is upregulated by EZH2, forming a positive feedback circuit involving FAK-GLI-1 and contributing to drug resistance, cancer stem cell survival and EMT. Taken together, the results suggest integrin α11 as a promising prognostic marker and a powerful therapeutic target for drug-resistant breast cancer.

**Supplementary Information:**

The online version contains supplementary material available at 10.1186/s13058-024-01827-4.

## Background

Breast cancer is the most common cancer in women worldwide, and the second leading cause of cancer death in women in the United States [1, 2]. The majority of breast cancers, nearly 70%, are hormone receptor-positive (ER+), while the remaining 30% are human epidermal growth factor receptor 2 positive (HER2+) and triple-negative breast cancer (TNBC) [3, 4]. Despite advances in treatment options for breast cancer patients, including personalized medicine based on targeted therapies, innate or acquired drug resistance remains a major hurdle to the effective treatment of breast cancer [5, 6], leading to recurrence and metastasis, affecting the overall survival of breast cancer patients.

Breast cancers will eventually develop resistance to drugs of different modalities [7–12] with diverse mechanisms of resistance, including expression of multidrug-resistant efflux pumps or enhancement of growth signaling molecules [13]and anti-apoptotic proteins [14–17]. However, regardless of the action mode of applied drugs, the acquisition of drug resistance involves a common mechanism, the survival and expansion of drug-refractory cancer stem cells (CSCs) [18, 19]. In addition to the therapy-resistance, CSCs also possess self-renewal, stem cell plasticity, anchorage independence, and migratory capabilities [20, 21], which play a critical role in the recurrence and metastasis after chemotherapy [22]. Because elimination of CSCs will be a key therapy for the treatment of advanced drug-resistant tumors, it is essential to identify key molecules that promote CSC survival and expansion.

The integrin family which is comprised of 18 α and 8 β integrin subunits plays important roles in normal development as well as tumor progression and metastasis by controlling many cellular functions, including proliferation and cell survival. The role of integrins in the CSC expansion associated with intrinsic and acquired resistance has been highlighted in various cancers including breast cancer [21, 23–27]. Among the integrin family, integrin α6 is known to promote the self-renewal ability of breast cancer cells through focal adhesion kinase (FAK) signaling linked to the expression of the Hedgehog effector GLI-1 and a key stem cell factor BMI-1 [28]. In addition, overexpression of integrin α6 was identified in tamoxifen-resistant breast cancer, and its inhibition has been shown to suppress tamoxifen resistance [29]. In TNBC cells, integrin α2 regulates cancer stemness phenotypes, such as stem cell marker expression, mammosphere formation, and FAK activation, which consequently leads to metastasis of cancer [30]. Similarly, integrin α9 knockdown abolished TNBC stem cell characteristics [31]. In addition, integrin β1 and β3 also enhance CSCs properties and chemoresistance of breast cancer cells [32, 33]. Notably, integrin β1 is also expressed in stem cells of normal tissues, but its expression level is increased in CSCs of chemo-resistant and metastasizing breast cancer cells [32, 34, 35]. Similarly, overexpressed integrin α3 and β4 play a key role in CSC survival and metastasis in androgen-refractory prostate cancer [36]. While several integrin subunits have been implicated in CSC survival and drug resistance across different cancer types, it remains to be determined whether there exists a master integrin subunit commonly involved in the development of resistance to multiple types of anti-cancer drugs.

In the present study, we identified integrin α11 as a key molecule governing CSC survival and resistance in tamoxifen- and doxorubicin (adriamycin)-resistant breast cancer cells, and elucidated the regulatory mechanisms of integrin α11 overexpression and its regulatory action in the survival of drug-resistant cancer cells and differentiation transition into mesenchymal cells.

## Methods

### Cell culture

MCF-7 was purchased from the American Type Culture Collection (Manassas, VA, USA). Tamoxifen-resistant MCF-7 cells (TAMR) and adriamycin-resistant MCF-7 cells (ADR) were generous gift from Prof. Keon Wook Kang (Seoul National University, Republic of Korea). MCF-7 cells were cultured in DMEM medium supplemented with 10% FBS and 1% penicillin/streptomycin whereas ADR and TAMR cells were cultured in DMEM medium supplemented with 10% FBS, 1% P/S and 3 µM doxorubicin and tamoxifen, respectively. All cells were incubated at 37 °C in a 5% CO_2_ atmosphere.

The passage (P) numbers of cells utilized for in vitro studies were as follows: P28 to P106 for MCF-7 cells, P42 to P84 for adriamycin-resistant (ADR) cells, P47 to P83 for tamoxifen-resistant (TAMR) cells, P5 to P10 for SUZ12-overexpressing (OV) cells, and P2 to P10 for EZH2-OV cells.

### Stable cell line generation

MCF-7 cells stably overexpressing SUZ12 or EZH2 were generated by transfecting MCF-7 cells with 5 µg SUZ12 (Cone ID: TRCN0000475819) or EZH2 plasmid (Clone ID: TRCN0000467064) (Mission shRNA plasmid DNA, Sigma-Aldrich) in the presence of lipofectamine 3000 (Invitrogen, Carlsbad, CA, USA), following the protocol outlined in the technical bulletin of MISSION lentiviral transduction particles (Sigma-Aldrich). After 48 h of transfection, transfected cells were selected by culturing with DMEM medium supplemented with 10% FBS and 2 µg/mL puromycin. Confirmation of the overexpression of each target gene was achieved through immunoblotting analysis.

The generation of luciferase-expressing TAMR cells followed the protocol outlined in the Luciferase Plasmid Guide (Addgene). Briefly, 5 µg of pCAG-Luciferse plasmid DNA (55,764, Addgene) was transfected to TAMR cells along with Lipofectamine 3000 (Invitrogen). Luciferase-expressing cells were selected by culturing them at geneticin (10 mg/mL)-containing media.

### Transcriptome and bioinformatics analysis

mRNAs were extracted and analyzed using the Nanostringn Ncounter Pancancer Pathway array kits and systems, provided by PhileKorea (Seoul, South Korea). Genes showing ≥ 2-fold change (*p* < 0.05) were considered differentially expressed genes (DEG) and were analyzed using Panther 16.0 software [37], followed by GO Enrichment Analysis tool (http://geneontology.org/) to validate the GO terms associated with Epithelial-Mesenchymal Transition (EMT), and StemChecker (http://stemchecker.sysbiolab.eu/) [38] to predict the stemness signature molecule.

To analyze the association between the expression of ITGA11 or EZH2, and the overall survival (OS) and relapse-free survival (RFS) in breast cancer patients, the Kaplan‑Meier‑plotter (KM plotter, http://kmplot.com/analysis/) was utilized. The cohort was classified based on high and low expression levels and with an autoselect best cutoff. The gene expression profiles of GSE20711, GSE20685, and GSE3494 were obtained from the NCBI Gene Expression Omnibus (GEO) database.

### siRNA transfection

siRNA transfection followed the siTran siRNA transfection application guide. Briefly, cells were seeded in antibiotic-free media and transfected with 100 nM siRNA targeting ITGA11, ITGB1, EZH2, SUZ12, HIF-1α, or GLI-1 (ORIGENE, Rockville, MD, USA) for 72 h using DharmFECT reagent 1 (Thermo Scientific, Waltham, MA, USA).

### Cell viability assay

Cell viability was measured by 3-(4,5-dimethylthiazol-2-yl)-2,5-diphenyltetrazolium bromide (MTT) assay [39]. Cells were seeded in 96-well plates in culture media containing 1% FBS for 24 h. Then, the cells were treated with vehicle, tamoxifen, or doxorubicin at the indicated concentrations. After 48 h, MTT solution (Merck, Burlington, MA, USA) was added. After 4 h of incubation, the media with MTT solution was removed and dimethyl sulfoxide (DMSO) was added to dissolve the formazan crystal. Absorbance was measured at 540 nm using a microplate reader (BMG LABTECH GmbH, Ortenberg, Germany).

### Measurement of apoptosis and caspase 3 activity

Apoptosis was measured using the FITC Annexin V apoptosis detection kit (BD Biosciences, San Jose, CA, USA) following to the manufacturer’s instructions. Briefly, cells treated with 100 nM non-target siRNA (siNT) or ITGA11 siRNA (si*ITGA11*) for 72 h were trypsinized and washed with ice-cold PBS. Thereafter, 1 × 10^5^ cells were suspended in 1× binding buffer (100 µL) and were stained with propidium iodide (5 µL) and Annexin V-FITC (5 µL) and incubated in the dark for 15 min at 25 °C. After that, 400 µL of 1× binding buffer was added and analyzed by flow cytometry (FACSVerse Cytometer, BD Biosciences, San Jose, USA).

Caspase-3 activity assay was performed using a caspase-3 assay kit (Abcam, Cambridge, MA, USA) following the manufacturer’s instructions. Cells transfected with siNT or si*ITGA11* were treated either with tamoxifen or doxorubicin (10 µM for 48 h). Then, cells were lysed and quantified for total protein using BCA protein assay reagent (Pierce; Rockford, IL, USA). Samples containing 200 µg of total protein were assayed for caspase-3 activity with DEVD-pNA (200 µM) as a caspase-3-specific substrate. Samples were incubated at 37 °C for 120 min and absorbance was measured at 405 nm using a microplate reader (BMG LABTECH).

### Western blot analysis

Cells were lysed with radioimmunoprecipitation assay (RIPA) buffer (Thermo Scientific, Waltham, MA, USA) containing 1X protease and phosphatase inhibitor cocktail (Thermo Scientific) for total protein extraction. Nuclear and cytoplasmic proteins were extracted using NE-PER nuclear and cytoplasmic extract reagents (Thermo Scientific), respectively. The proteins separated by SDS-PAGE were transferred to a nitrocellulose membrane (Whatman GmbH, Dassel, Germany) and subjected to immunoblotting with primary and secondary antibodies. Immunoblots were visualized using an ECL kit (Thermo Scientific). Images were captured using the LAS-4000 mini system (Fuji, Tokyo, Japan).

### Co-immunoprecipitation assay (co-IP)

Immunoprecipitation was conducted using a 100 µg of total proteins, employing an IP-grade Integrin α11 antibody or IgG (1 mg/mL) (Sigma-Aldrich), incubated for 16 h at 4 °C. Subsequently, Protein A agarose beads (50 µL) (Thermo Scientific) were added in the immunoprecipitated solution and allowed to incubate for 4 h at 4 °C. The resultant immune complexes were collected through centrifugation at 3000× g for 2 min at 4 °C. The collected pellet was washed with PBS and then re-suspended in 25 µL of 1× sample buffer (62.5 mM Tris-HCl pH 6.8, 2.5% SDS, 0.002% Bromophenol Blue, 0.7135 M (5%) β-mercaptoethanol, 10% glycerol), followed by heating at 95 °C for 5 min. After centrifugation at 12,000× g for 30 s at 4 °C, supernatant containing the IP samples was collected.

### Sphere formation assay

Cells (1 × 10^3^) were seeded onto 24-well low attachment plates (Corning Costar, Corning, NY, USA) in prEGM media (Lonza, Basel, Switzerland) and grown in spheroids. After 15 days, an inverted microscope (TE2000-U; Nikon, Tokyo, Japan) was used to capture images of the spheres. The number of spheroids larger than 50 μm in diameter was counted using Image J 1.48v software (National Institute of Health, Bethesda, MD, USA).

### Flow cytometry analysis for stem cell population

Single cell suspension (1 × 10^7^ cells/mL) in cold PBS containing 3% FBS were stained with APC-anti-human CD24, FITC-anti-human CD44, and their respective isotype control antibodies (APC-anti-mouse IgG 2a, and FITC-anti-mouse IgG1) for 30 min in the dark at 4 °C. Stained cells were washed twice and analyzed by flow cytometry (FACSVerse Cytometer, BD Biosciences).

### Immunocytochemistry

Breast cancer cells (1 × 10^5^ cells) were seeded on confocal dishes (SPL Life Science, Pocheon, Korea). After 24 h incubation at 37 °C, cells were fixed with 4% paraformaldehyde in PBS (pH 7.4) for 10 min at 25 °C. After fixation, cells were washed three times for 5 min with PBS and permeabilized with 0.1% Triton X-100 for 10 min at 25 °C. Cells were then incubated with 1% BSA (bovine serum albumin) in 1× PBST for 1 h to prevent non-specific binding. After blocking, cells were incubated with E-cadherin and Vimentin primary antibodies in 1% BSA in PBST overnight at 4 °C, stained with an Alexa fluor 488 anti-mouse and Alexa fluor 647 anti-rabbit secondary antibody in the dark for 1 h at 25 °C, washed, counterstained with 1 µg/ml DAPI, and rinsed with PBS. Images were captured at 400X magnification using an inverted fluorescence microscope (TE2000-U; Nikon).

### Transcription factor (TF) activation profiling array

A Cancer Stem Cell TF Activation Profiling Plate Array (Signosis, Santa Clara, CA, USA) was used to compare the activity of stemness-associated TFs. In brief, nuclear lysates were treated with biotin-labeled probes containing consensus sequences of TF DNA binding sites for 30 min at 25 °C. The spin column purification method was used to separate the TF/probe complex mixtures. Using an elution buffer, the bound probes were released from the complex and centrifuged at 9,800 g for 2 min. After the eluents were denatured at 98 °C for 5 min, the denatured sample was added to TF hybridization buffer. The resulting mixture was then put to each well of the hybridization plate, the plate was sealed with aluminum adhesive and the hybridization plate was then incubated at 42 °C for 16 h. In order to find the bound DNA probe, a streptavidin-horseradish peroxidase conjugate was used. Endpoint luminescence readings of the samples were observed using Fluostar omega (BMG LABTECH, Ortenberg, Germany).

### Chromatin immunoprecipitation (ChIP) assay

Chromatin extraction and subsequent ChIP assay were performed using chromatin extraction kit (Abcam) and ChIP Kit-One Step (Abcam) according to manufacturer’s instructions. After chromatin cross-linking with 1% formaldehyde, chromatin was extracted and sheared by sonication. The lysates were incubated with antibodies against HIF-1α, GLI-1, EZH2, RNA Pol II or IgG for 2 hours at 25°C. Then, samples were digested using proteinase K, and DNA was subjected to qPCR using SYBR Green (Qiagen; Germantown, MD, USA) and primers (Bioneer; Daejeon, Korea). The primers used were ITGA11 (Forward 5′-CACGACATCAGTGGCAATAAG-3′ and Reverse 5′-GACCCTTCCCAGGTTGAGTT-3′), ITGB1 (5′-GCAAGCTCAGGCATAACAGC-3′ and 5′-CCCTGGCTCAGAGAGAATGC-3′), ITGB8 (5′-CTGTTTGCAGTGGTCGAGGAGT-3′ and 5′-TGCCTGCTTCACACTCTCCATG-3′), EZH2 (5’- CCCTGACCTCTGTCTTACTTGTGGA − 3’ and 5’-ACGTCAGATGGTGCCAGCAATA-3’ or GAPDH (5′-ACCACAGTCCATGCCATCAC-3′ and 5′-TCCACCACCCTGTTGCTGTA-3′). Input DNA (1%) was used for percentage binding analysis.

### Migration assays (wound healing and transwell migration)

For wound healing assay, TAMR or ADR cells transfected with siNT, siITGA11, siSUZ12 or siEZH2 were seeded in 24 well plates. Cells were scratched with sterile 10 µL disposable plastic pipette tips and washed with PBS. The cells were incubated with serum-free medium containing 10 µg/mL Mitomycin C (to inhibit cell proliferation). After 2 h, the cells were treated with 5% serum as an inducer. The wound healing procedure was observed by microscopy, and images were captured at 0 and 48 h after treatment with an inducer. The distance between the wound edges was measured using Image J 1.48v software.

For transwell migration assay, 1 × 10^6^ cells per well were seeded in the upper chamber (8.0-µm pore membrane) in 100 µL of 1% FBS containing medium, and 600 µL of 5% FBS containing medium was added to the bottom chamber. After 18 h incubation at 37 °C in a 5% CO_2,_ the cells on the upper surface of the membrane were removed with cotton swab, and the migrated cells on the lower surface of the membrane were fixed using methanol, and stained with hematoxylin and eosin. The numbers of cells migrated per field were captured at 200× magnification using a digital camera fitted to the inverted microscope (Nikon, Tokyo, Japan). The captured images were used to count the number of migrated cells.

### Animal experiment ethics

The animal experiments (chick embryo and mouse experiments) were approved beforehand by the Institutional Animal Care and Use Committee (IACUC) of Yeungnam University and were performed following the institutional guidelines of the Institute of Laboratory Animal Resources (1996) and Institutional Animal Care and Use Committee of Yeungnam University (2009).

### Measurement of anti-tumor and anti-metastatic activity using chick chorioallantoic membrane (CAM)

Fertilized eggs were procured from Byeolbichon Farm (Gyeongbuk, South Korea), and eggs were incubated at 37 °C in 55% relative humidity. A small hole was made using a hypodermic needle on the wider side of 9-day-old fertilized eggs after selection of bifurcated vessels. Another hole was made on the broad side by applying negative pressure to the first hole and creating a false air sac that was later sealed. A window (1 cm^2^) was made above the false air sac. MCF-7, siRNA (NT, SUZ12, EZH2, or ITGA11) transfected TAMR or ADR cells (1.5 × 10^6^ cells/CAM) were inoculated on the CAM. Eggs were returned to the incubator after sealing the window. CAM tissue beneath the tumor region was resected from the embryo and then harvested. Blood vessels in the tumor region were viewed using an optical microscope (Olympus Corporation, Tokyo, JAPAN) and counted. Tumor tissues detached from the CAM were weighed.

For metastatic study, 1.5 × 10^6^ cells/CAM labelled with cell-tracking red-florescent dye were mixed in 50% Matrigel and implanted on the exposed CAM. On the 5th day of implantation, the lower CAM and liver of developing chicken embryo were collected to evaluate metastatic cells using fluorescence-aided Leica L2 microscope (Leica, Tokyo, Japan). The lower CAM and liver tissues were further analyzed to detect human DNA hypoxanthine phosphoribosyl transferase (HPRT) using PCR.

P34 and P48 for MCF-7, P54 and P62 for ADR, and P58 and P64 for TAMR were used for inoculation onto the CAM.

**Bioluminescence imaging and survival rate measurement in a mouse tumor model.**

Seven weeks-old female BALB/c nude mice (OrientBio, Gyeonggi, South Korea) were injected subcutaneously with 1.5 × 10^6^ TAMR-Luc cells (P5) in 200 µL of DMEM/Matrigel (1:1) into the right flank. Tumors were allowed to grow untreated until they reached approximately 200 mm^3^. A total of 20 tumor-bearing nude mice were randomly divided into 4 groups and were used to evaluate the efficacy of treatment. The mice were then administered twice a week for 2 weeks with siRNA complex targeting none (siNT), integrin α11 (siITGA11), or EZH2 (siEZH2) by intratumoral injection. Each siRNA complex contained 5 µg of siRNA and 3.5 µL Oligofectamine (Invitrogen). Tumor size was calculated using the equation (l × b^2^)/2, where l and b were the larger and smaller dimensions of each tumor, respectively. In addition, the body weights of the mice were also measured to evaluate tumor progression degree. For in vivo imaging of the tumor at the 14th day of the first drug administration, mice were administered with D-luciferin (150 mg/kg) (Promega, Madison, WI) by intraperitoneal injection and placed in a light-tight mouse imaging chamber (Xenogen IVIS Imaging System, Xenogen, US) under anesthesia of 2.5–3% isoflurane. At 10 min after D-luciferin injection, the converted D-luciferin was measured in the value of emitted photons using Living Image software 4.0 (Caliper, Alameda, CA.). Afterward, the mice were returned to their cages, and the survival rate was measured.

### Statistical analysis

Data from more than three independent experiments were averaged. Data are expressed as mean ± SEM. Statistical significance was assessed by one-way analysis of variance (ANOVA) followed by Newman-Keuls comparison using Graph Pad Prism 8.0 (San Diego, CA, USA). p values lower than 0.05 were considered significant.

## Results

### Integrin α11 overexpression is essential for drug-resistant breast cancer cell survival

**Fig. 1:**
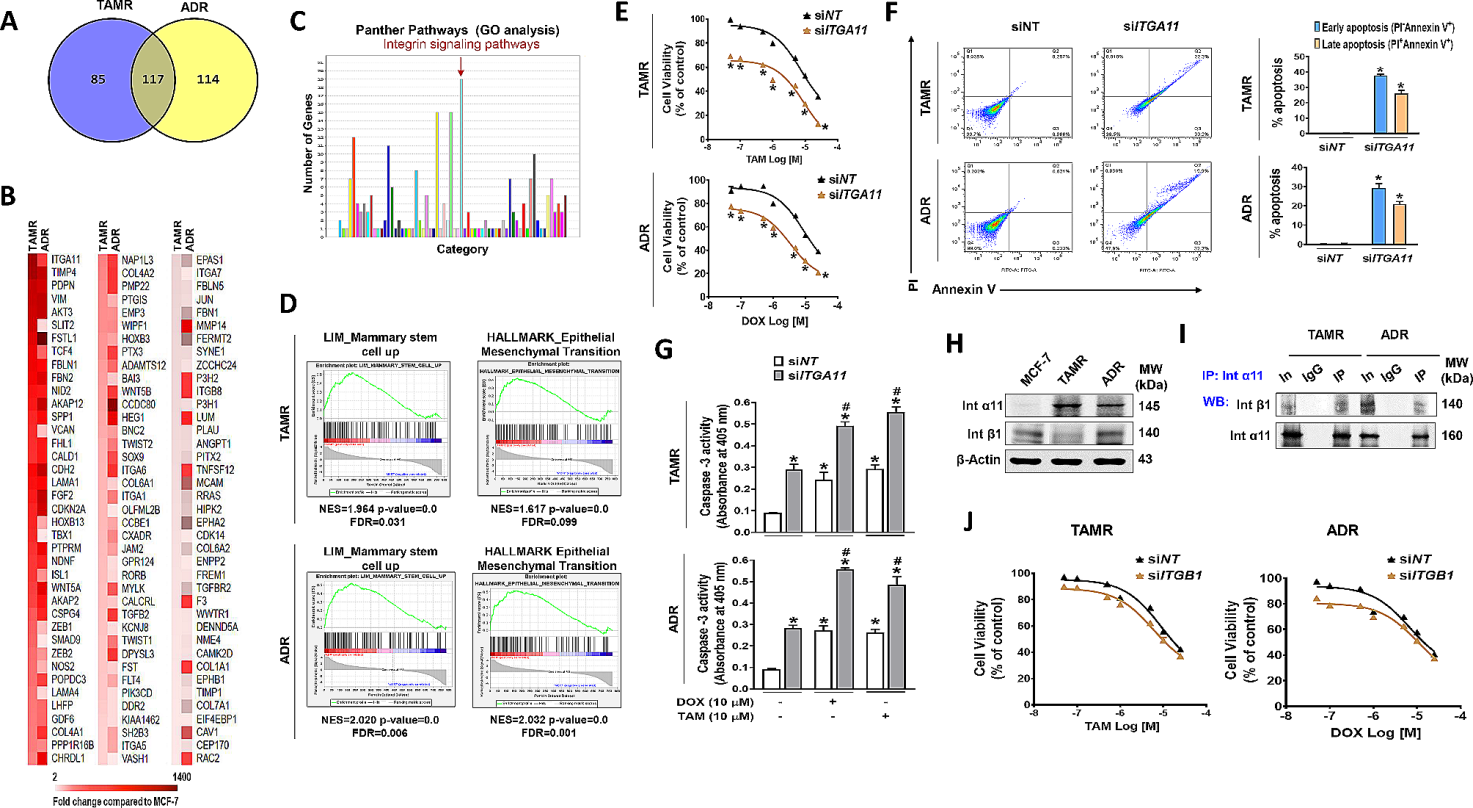
**Integrin α11 overexpression induces resistance to anticancer drugs in breast cancer cells** (A) Differential gene expression analysis was conducted using Nanostring nCounter Pancancer Pathway array kits, and the results were visualized as a Venn diagram. (B, C) The 117 genes commonly up-regulated in TAMR and ADR compared to parental MCF-7 cells are shown as heatmaps (B) and functionally enriched Gene Ontology (GO) pathways (fold change ≥ 2, *p* < 0.05) (C). (D) Gene Set Enrichment Analysis (GSEA) of transcriptome data was performed on TAMR and ADR cells. (E-G) Transfection of TAMR and ADR cells with *ITGA11* siRNA recovers sensitivity to tamoxifen or Doxorubicin: Cell viability by MTT assay (E), FACS analysis showing early and late apoptosis (F), and caspase-3 activity (G) in *ITGA11* siRNA-transfected TAMR and ADR cells in the presence of different concentrations of tamoxifen or Doxorubicin. *p < 0.05, compared to siNT-transfected group. ^#^*p* < 0.05 compared to siRNA and vehicle-treated group. (H) Western blot analysis shows the expression of Integrin α11 and integrin β1 at basal level of MCF-7, TAMR, and ADR cells. (I) Western blots of immunoprecipitates (IP) with an anti-integrin α11 antibody show that integrin α11 interacts with integrin β1. IgG was used as a negative control antibody. ‘In’ represents input (the total protein lysate used). (J) TAMR and ADR cells transfected with *ITGB1* siRNA were analysed for recovery of sensitivity to tamoxifen and Doxorubicin

To identify the common key molecules regulating the survival of drug-resistant breast cancer cells, we examined differentially expressed genes in two different drug-resistant breast cancer cell lines, tamoxifen-resistant (TAMR) and adriamycin-resistant (ADR) MCF-7 cell lines. Our transcriptome data analysis revealed that out of 740 genes (Supplementary Fig. S1A), 202 and 231 genes were differentially upregulated with statistical significance in TAMR and ADR cells, respectively, compared to the MCF-7 parental cells (Fig. 1A). The commonly upregulated 117 genes in both TAMR and ADR cells (Fig. 1B) were highly associated with integrin signaling pathway in Protein Analysis through Evolutionary Relationships (PANTHER) pathway analysis (Fig. 1C). Among the significantly altered integrin genes, *ITGA11* was commonly up-regulated in both cell lines, with TAMR cells showing the higher expression, while ADR cells showed a moderate increase (Fig. 1B; Table 1). The gene set enrichment analysis (GSEA) to further extract the other enriched mechanistic pathways in the two sets of total genes showed significant enrichment of genes in mammary stem cells and EMT in TAMR and ADR cells (Fig. 1D, Supplementary Fig. S1B). The PANTHER and GSEA analysis findings indicate that integrin overexpression is a crucial signal regulating CSC growth and acquired drug resistance in breast cancer cells. To verify this, we first examined the effect of silencing highly increased gene *ITGA11* on the survival of TAMR and ADR cells in response to anticancer drugs. Despite the expression level difference in the two cell lines, knock-down (KD) of *ITGA11* potentiated the drug-induced cell viability decrease in both TAMR and ADR cells (Fig. 1E). The decreased cell viability by *ITGA11* siRNA (si*ITGA11*) treatment coincided with increased apoptosis: si*ITGA11*-transfection enhanced the drug (tamoxifen or Doxorubicin)-induced early and late apoptosis populations (Fig. 1F) and the activities of caspase 3, the apoptosis executioner [40] in both TAMR and ADR cells (Fig. 1G). Because integrin α11 is reported to dimerize with integrin β1 [41, 42], we also examined the level of dimerization intensity and role of integrin β1 in the survival of the resistant cell lines. Compared to the parental cell line MCF-7, integrin β1 expression was rather lower in TMAR cells but was slightly increased in ADR cells, which is opposite to the extent of integrin α11 expression in the two cell lines (Table 1; Fig. 1H). Despite the levels, integrin α11 formed a heterodimer with integrin β1 evidenced by co-immunoprecipitation (Fig. 1I). However, *ITGB1* KD did not alter the drug-induced cell death in both TAMR and ADR (Fig. 1J), indicating that integrin β1 did not play a functional role in drug-induced cell death.

**Table 1:** Integrin gene expressions with significant difference in TAMR and ADR cells compared to MCF-7 cells

	TAMR		ADR	
	Fold Change^a)^	P-Value	Fold Change^a)^	P-Value
ITGA11	665.23	0.0004	31.47	0.0139
ITGA6	9.87	0.0022	51.68	0.0025
ITGA1	9.24	0.0074	2.95	0.0018
ITGA8	6.48	0.0097	-2.44	0.0173
ITGA5	5.7	0.0327	11.66	0.0069
ITGA7	5.16	0.0052	2.56	0.0399
ITGB8	4.59	0.0223	42.97	0.0014
ITGB3	2.01	0.1919	2.78	0.0731
ITGA9	1.85	0.3860	1.07	0.9092
ITGB2	1.5	0.5898	1.95	0.3875
ITGB7	1.3	0.2244	1.18	0.3891
ITGB1	-2.55	0.006	1.7	0.1330
ITGB6	-4.47	0.0814	-1.47	0.5835
ITGA3	-4.92	0.0211	10.27	0.0190
ITGA2	-12.71	0.0001	-19.8	0.0023
ITGB4	-20.76	0.0216	6.24	0.0307

^a)^ The gene expression level was compared to that of parental MCF-7 cells

### Integrin α11, a key molecule for CSC enrichment, is regulated by EZH2

**Fig. 2:**
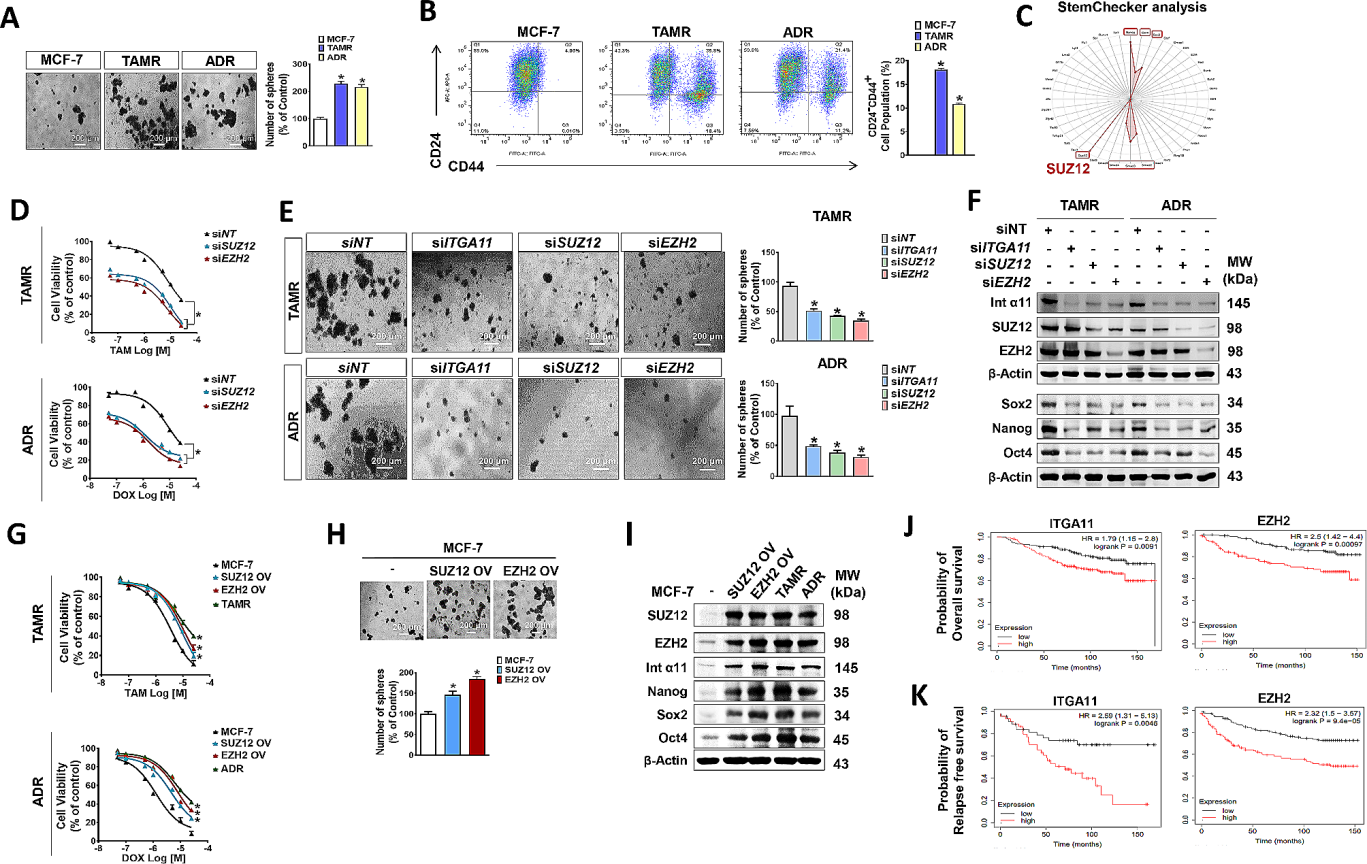
EZH2-mediated integrin α11 overexpression in drug-resistant breast cancer increases cancer stem cell populations (A) Enhanced spheroid formation was observed in TAMR and ADR cells. The scale bar (white) corresponds to 200 μm. The bar graph quantifies spheroids with diameters exceeding 50 μm. (B) Analysis of cancer stem cell populations within MCF-7, TAMR, and ADR cells. The bar graph shows the relative abundance of CSCs (CD24-CD44+) from three independent experiments. (C) In silico examination of common DEGs to predict shared stemness-associated molecules in TAMR and ADR cells using the StemChecker online tool. (D, G) Effects of silencing (D) and overexpression (G) of SUZ12 or EZH2 on the viability of cells treated with tamoxifen or doxorubicin. (E, H) Sphere-forming ability of cancer cells transfected with siRNAs (E) or overexpressing plasmids (H) of EZH2 or SUZ12. (F) Immunoblots reveal the effect of ITGA11, SUZ12, or EZH2 KD on the expression of integrin α11 and stemness marker proteins (Nanog, Sox2, and Oct4). (I) Comparison of the effects of SUZ12/EZH2 overexpression on integrin α11 expression and stemness marker proteins (Nanog, Sox2, and Oct4) with TAMR and ADR. (J) Overall survival rates of breast cancer patients with high and low expression of *ITGA11* (Left) and *EZH2* (Right). (K) Probability of relapse-free survival in breast cancer patients with high and low expression of ITGA11 (Left) and EZH2 (Right)

Considering that chemoresistance is one of the hallmarks of CSCs, we investigated whether the proportion of CSCs differs between the drug-resistant breast cancer cell lines and examined the involvement of integrin α11 in the CSC population regulation. TAMR and ADR cells showed a significant increase in CSC population, which was revealed by the sphere-forming ability (Fig. 2A) and relative number of CSCs that were identified with antibodies specific to cancer stem cell surface markers, CD24 negative and CD44 positive (CD24-/CD44+) (Fig. 2B). To identify the genes associated with CSC maintenance, we further analyzed the genes that are commonly upregulated in both TAMR and ADR cells with Stem Checker program. Stem Checker analysis revealed highly significant enrichment of stem cell-related genes, Nanog, Oct4, Sox2, Smad2/3/4, and SUZ12, of which SUZ12 was the most significant molecule (Fig. 2C). SUZ12 is a protein belonging to Polycomb group (PcG) and forms the core essential subunits of the Polycomb Repressive Complex 2 (PRC2) with EZH2 and EED for the regulation of various gene expression [43, 44]. Silencing SUZ12 or its catalytic subunit EZH2 induced an increase in tamoxifen- and doxorubicin-induced cell death (Fig. 2D) and a decrease in CSC population (Fig. 2E) in both TAMR and ADR cells. KD of SUZ12 or EZH2 significantly downregulated the expression of stemness-associated genes and integrin α11, the extent of which was similar to the effect of integrin α11 KD, except that KD of ITGA11 did not change the expression of EZH2 and SUZ12 (Fig. 2F, Supplementary Fig. S2A). The results indicate that EZH2/SUZ12 acts as an upstream regulator of ITGA11. Conversely, overexpression (OV) of SUZ12 or EZH2 in parental MCF-7 cells induced resistance to the drugs, and the extent was similar to the response pattern of TAMR and ADR cells to the drugs (Fig. 2G). Also, the SUZ12- or EZH2-overexpressed MCF-7 cells enhanced CSC population (Fig. 2H). Corresponding to the phenotype changes, expressions of stemness-associated genes (Nanog, Oct4, and Sox2) and integrin α11 were enhanced by OV of SUZ12 or EZH2 in MCF-7 cells, similar degree to the resistant cells (Fig. 2I, Supplementary Fig. S2B). The significance of the overexpression of *EZH2* and *ITGA11* in cancer progression and aggressiveness was also confirmed in breast cancer patients, using Kaplan-Meier survival analysis on The Cancer Genome Atlas (TCGA) dataset. High-level expression of ITGA11 or EZH2 was associated with poor survival (Fig. 2J) and a decrease in recurrence-free survival (Fig. 2K) of breast cancer patients.

### EZH2 cooperatively interacts with HIF-1α and FAK-activated GLI-1 for the regulation of integrin α11 expression

**Fig. 3:**
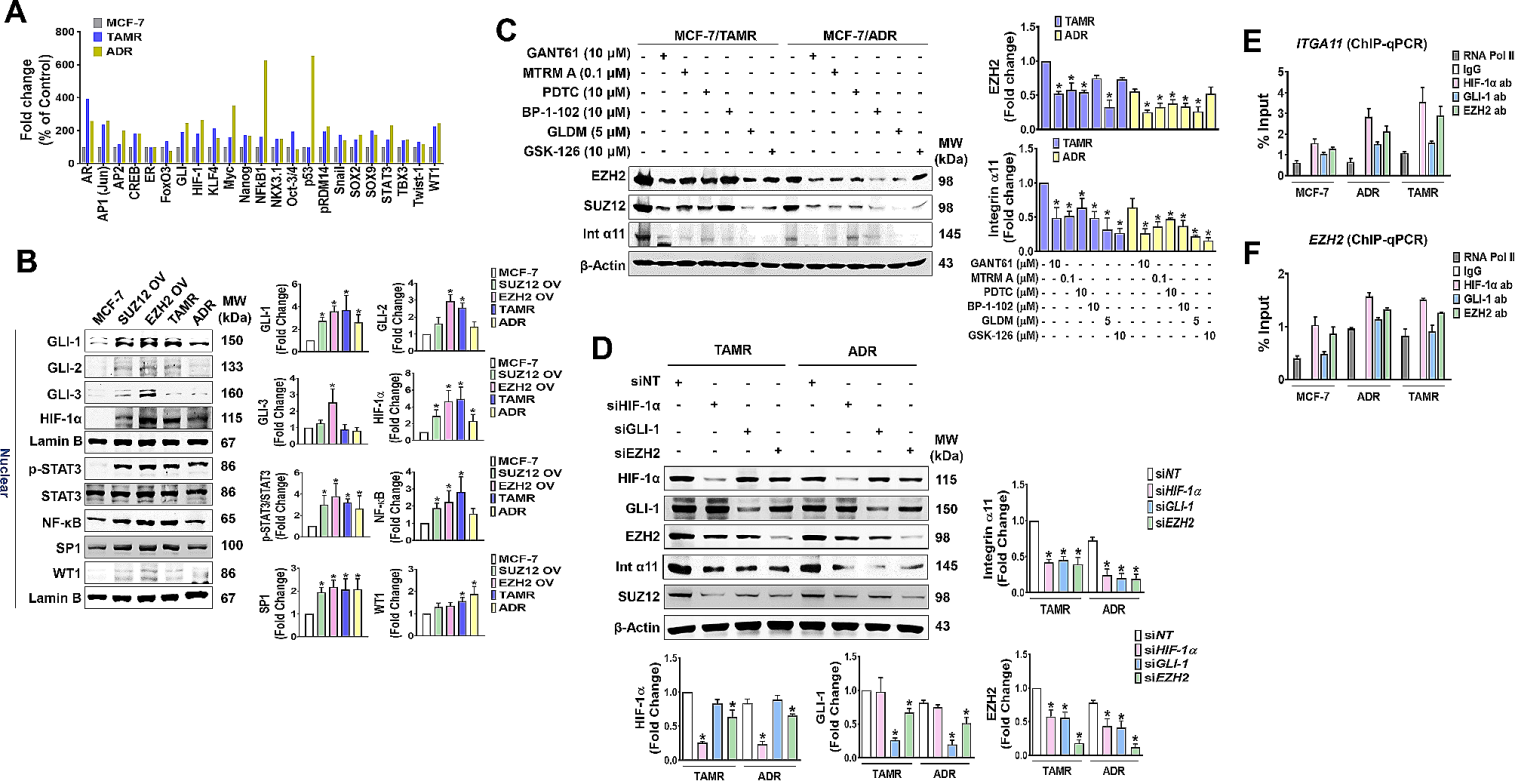
**HIF-1α/GLI-1/EZH2 are the major TFs for EZH2-integrin α11 axis overexpression** (A) Cancer stem cell transcription factor activating profiling plate array. The bar graph represents mean SEM of three independent experiments. (B) Comparison of TF expression levels between the EZH2- or SUZ12-OV and drug-resistant cells. Fold change values are derived from three independent experiments. (C) The effects of various TF inhibitors on the expression of EZH2, SUZ12, Integrin α11 in TAMR or ADR cells. MTRM A, GLDM, and Int represent mithramycin A, geldanamycin, and integrin, respectively. (D) Comparison of integrin α11 expression levels by silencing TFs, HIF-1α, GLI-1, and EZH2 in TAMR or ADR cells. *p < 0.05 compared to siNT-transfected group. (E, F) ChIP and qPCR validation demonstrating HIF-1α, GLI-1, or EZH2 binding to ITGA11 (E) and EZH2 (F). Negative control (IgG) and positive control (RNA polymerase II) were used

Because both EZH2 and ITGA11 genes supporting CSCs were overexpressed in the resistant cells, a cancer stem cell TF activation profiling array was conducted to identify regulatory transcription factors (TFs) responsible for the genes. Various stemness-associated TFs were activated in the resistant cell lines, with nuclear factor-κB (NF-κB) and p53 showing exceptionally high levels only in ADR cells (Fig. 3A). This increase pattern was similarly observed in EZH2/SUZ12-OV cells, showing an overall increase in nuclear (active) levels of the TFs (Fig. 3B). Among them, GLI-1 was consistently higher in the SUZ12/EZH2-OV and drug-resistant cells (Fig. 3B). Similarly, nuclear HIF-1α and p-STAT3 were also consistently higher in the four variants of MCF-7 cells. However, the nuclear levels of NF-κB, SP1, and WT1 were somewhat different among the four cell types: NF-κB and SP1 levels were significantly increased in SUZ12/EZH2-OV and TAMR cells, but not in ADR cells, while nuclear WT1 level was significantly increased only in the drug-resistant cells (Fig. 3B). To investigate the relative contribution of these TFs in integrin α11 and EZH2 gene expressions, chemical inhibitors of the TFs were used. The expression of EZH2/SUZ12 in TAMR cells was significantly inhibited by GANT61 (GLI-1 inhibitor), mithramycin A (SP1 inhibitor), PDTC (NF-kB inhibitor), and geldanamycin (HIF-1α inhibitor), but not by BP-1-102 (STAT3 inhibitor) and GSK-126 (EZH2 inhibitor), while the gene expression in ADR cells was significantly inhibited by the inhibitors except GSK-126 (Fig. 3C). The strongest inhibition of EZH2/SUZ12 expression was achieved by geldanamycin and GANT61 in TAMR and ADR cells, respectively (Fig. 3C). Integrin α11 gene expression was inhibited by all the inhibitors used in both TAMR and ADR cells, with GSK-126 showing the strongest inhibition (Fig. 3C). The results indicate that GLI-1 and HIF-1α regulate EZH2 expression, which in turn regulate integrin α11 expression (Fig. 3C). The regulatory role of these TFs in EZH2 and integrin α11 expressions was further confirmed by silencing each of the TFs. Treatment of the resistant cells with siRNA specific to GLI-1, HIF-1α, or EZH2 showed that integrin α11 expression was reduced equally by all three siRNAs (Fig. 3D). In contrast, the effects of the siRNAs on EZH2 expression were different, with EZH2 knockdown being more than twice as potent as GLI-1 and HIF-1α knockdown (Fig. 3D). We further examined that these TFs are binding to ITGA11 and EZH2 by performing chromatin immunoprecipitation (ChIP)-qPCR analysis with specific antibodies to GLI-1, HIF-1α, and EZH2. All three TFs bound to the promoter of ITGA11, in the order of HIF-1α, EZH2, and GLI-1 (Fig. 3E). For EZH2, the binding of the three TFs to the gene promoter was lower than for ITGA11, but the degree of binding among the TFs was similar to ITGA11 (Fig. 3F).

**Fig. 4:**
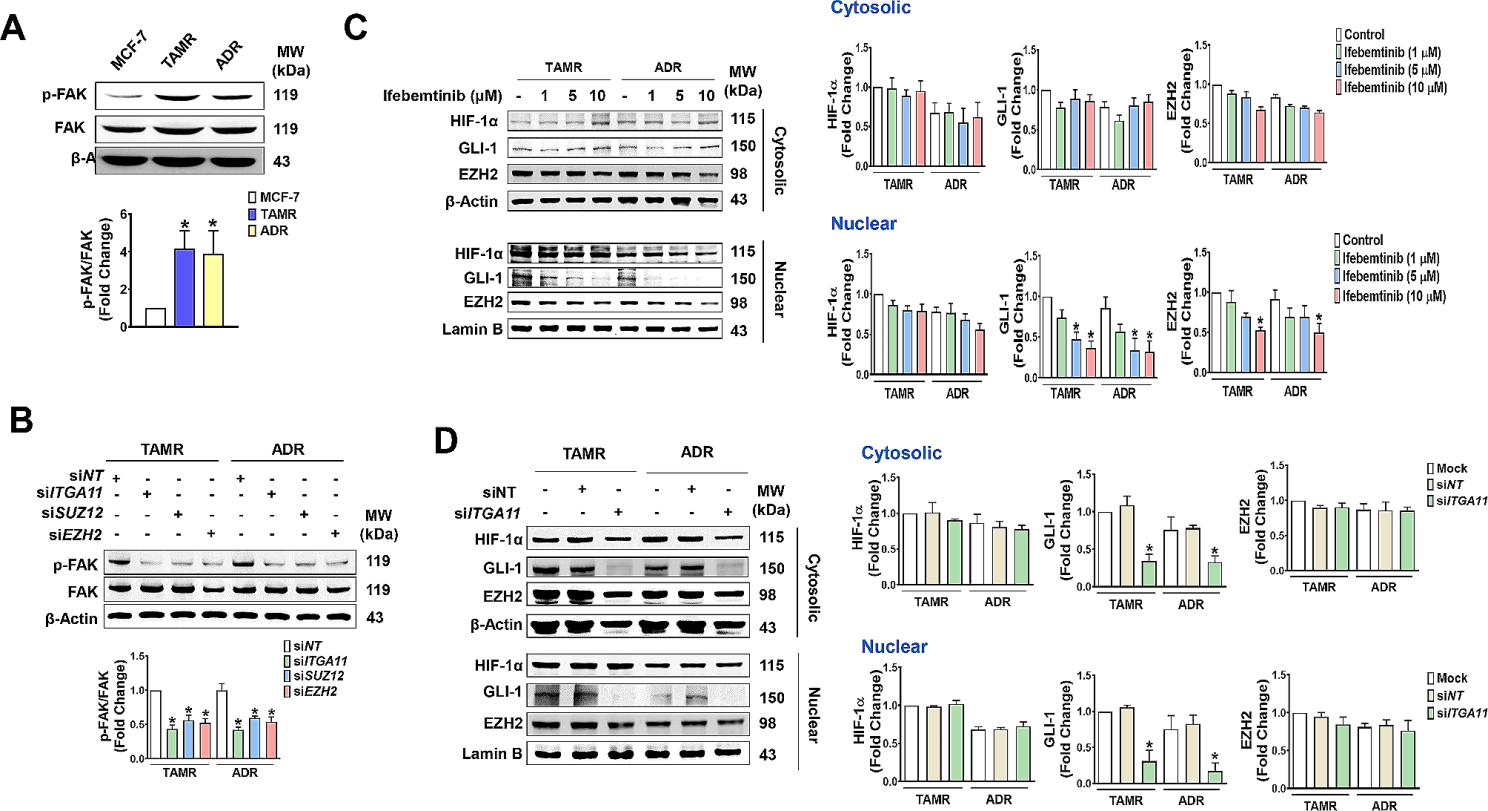
**GLI-1 is the critical component for positive feedback regulation of EZH2-integrin α11 axis** (A) Increased level of p-FAK in TAMR and ADR cells. *p < 0.05 compared to parental MCF-7 cells. (B) The effects of ITGA11, SUZ12, and EZH2 KD on FAK activity in TAMR and ADR cells. (C) Ifebemtinib, a FAK inhibitor, inhibits the nuclear level of GLI-1, but not of HIF-1α, GLI-1 and EZH2, in a concentration-dependent manner. *p < 0.05 compared to vehicle-treated control group. (D) *ITGA11* KD inhibits both cytosolic and nuclear level of GLI-1. *p < 0.05 compared to siNT-transfected group

Because SUZ12 or EZH2 OV enhanced the expression of the TFs, and TF knockdown in the resistant cells regulated integrin α11 and EZH2 expression, the results suggest a feedforward interaction between the TFs and the EZH2-integrin α11 axis. As a first step to uncover the mechanism underlying the axis, we examined whether the reciprocal interaction is mediated through FAK, an integrin receptor signaling molecule. Activation of FAK was higher in the resistant cells (Fig. 4A). In the cells with EZH2 or integrin α11 KD significantly inhibited the phosphorylation of FAK (Fig. 4B). On the other hand, treatment of TAMR and ADR cells with the FAK inhibitor ifebemtinib had differential effects on the level of three TFs. Ifebemtinib significantly inhibited nuclear translocation of GLI-1 in a concentration-dependent manner. However, cytosolic and nuclear HIF-1α levels were not significantly altered, while nuclear EZH2 levels were only significantly inhibited by a high concentration of ifebemtinib (Fig. 4C). Similar results were observed in the cells treated with ITGA11 siRNA, where GLI-1 levels in the cytosol, as well as nucleus, were significantly inhibited, but levels of HIF-1α and EZH2 were unchanged (Fig. 4D). The results indicate that integrin α11 regulates GLI-1 expression, which serves the critical connecting molecule in the EZH2-integrin α11 axis overactivation.

### EZH2/SUZ12-Integrin α11 axis regulates epithelial to mesenchymal transition (EMT) and metastasis of breast cancer cells

**Fig. 5:**
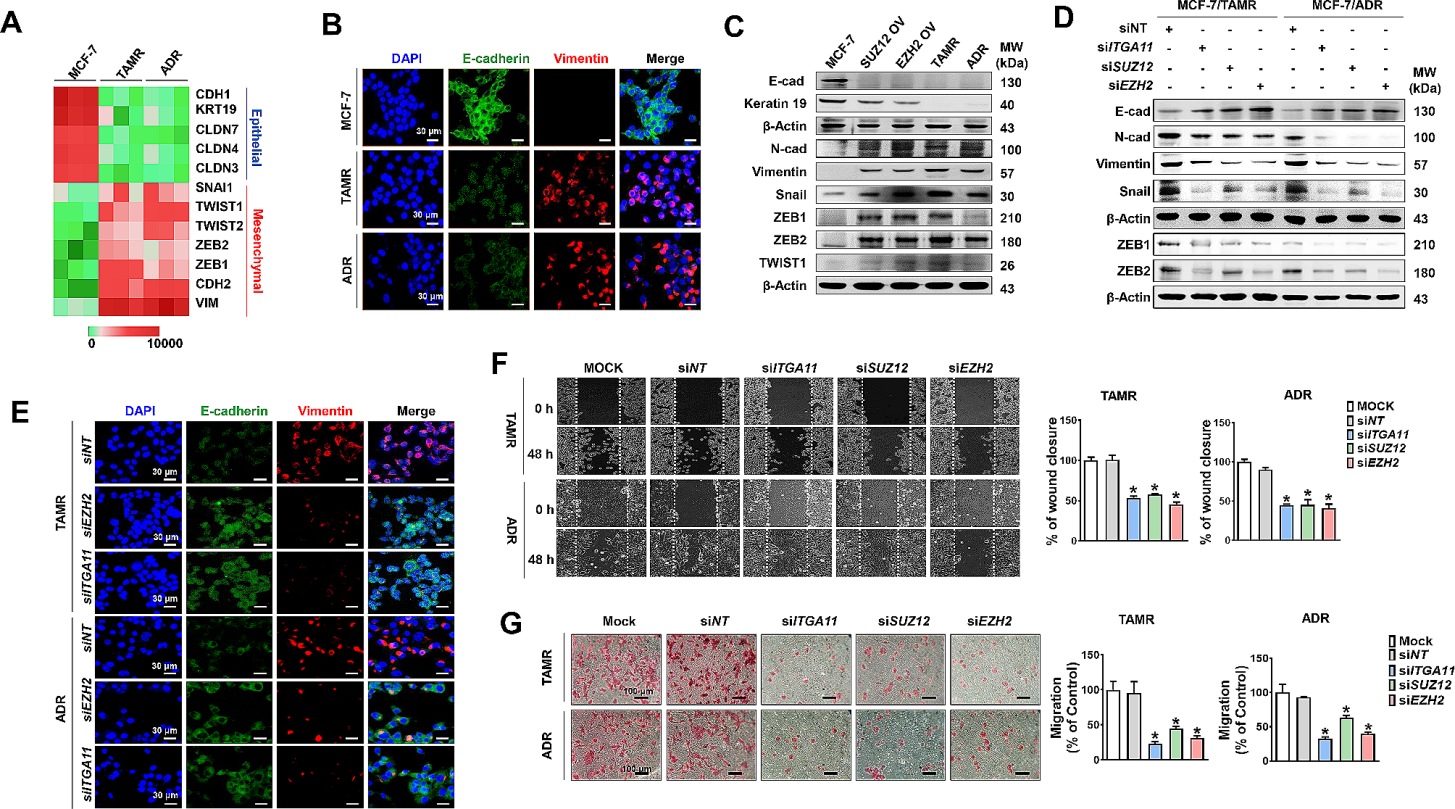
**The EZH2-Integrin α11 axis regulates EMT in drug-resistant breast cancer cells** (A) Heat map showing the expression of selected EMT-associated genes in TAMR and ADR cells compared to parental MCF-7 cells. (B) Comparison of E-cadherin and Vimentin expression levels in MCF-7, TAMR, and ADR cells stained with fluorescence-labelled antibodies. The scale bar (white) represents 30 μm at an original magnification of 400×. (C, D) Immunoblots demonstrating the effects of overexpression of SUZ12 or EZH2 (C) and silencing of ITGA11, SUZ12, or EZH2 (D) on the expression of epithelial (E-Cadherin, Keratin19) and mesenchymal cell markers (Vimentin, Snail, ZEB1, ZEB2, TWIST1). (E) Representative fluorescence microscopy images revealing E-cadherin and Vimentin expression patterns in TAMR and ADR cells treated with ITGA11 or EZH2. (F, G) KD of ITGA11, SUZ12, or EZH2 inhibits migratory ability of TAMR and ADR cells, revealed by wound healing assay (F) and transwell migration assay (G). The graphs represent the quantification of wound closure measuring the distance between wound edges using Image J software in the wound healing assay, and the number of migrated cells in the transwell migration assay. The scale bar (black) represents 100 μm at an original magnification of 200× for the transwell migration assay. *p < 0.05 compared to siNT-transfected group

Next, because the GSEA analysis and transcriptome data of TAMR and ADR revealed EMT of the drug-resistant cells, we also investigated whether the EZH2-integrin α11 axis is the key regulator of EMT, which plays multiple roles in cancer progression including drug resistance, metastasis, and recurrence [45–47]. Compared to MCF-7 cells, TAMR and ADR cells downregulated epithelial cell markers such as KRT19, CDH1, CLDN7, CLDN4, and CLDN3, and upregulated mesenchymal cell markers such as SNAI1, TWIST1, TWIST2, ZEB1, ZEB2, CHDH2, and VIM (Fig. 5A). The expression level of E-cadherin and vimentin, the representative markers for epithelial and mesenchymal phenotypes, respectively, were also confirmed by confocal microscopy (Fig. 5B) and immunoblotting (Fig. 5C). Such EMT gene expression patterns seen in drug-resistant cells were equally induced by OV of EZH2 or SUZ12 in MCF-7 cells (Fig. 5C, Supplementary Fig. S2C). Conversely, KD of EZH2, SUZ12, or ITGA11 in the resistant cells induced a reverted pattern of the gene expressions, which was revealed by immunoblotting (Fig. 5D, Supplementary Fig. S2D) and confocal microscopy (Fig. 5E). Similarly, EZH2/SUZ12 OV (Fig. 5C) and KD (Fig. 5D) induced increased and decreased expression of EMT-related transcription factors (TFs) such as Snail, ZEB1 and ZEB2, respectively. Corresponding to the gene expression changes, silencing the EZH2, SUZ12, or ITGA11 gene in the resistant cell lines resulted in a significant reduction in cell migration, which was assessed by scratch wound assay (Fig. 5F) and migration assay (Fig. 5G). These results indicate that the EZH2/SUZ12-integrin α11 axis regulated the genes associated with EMT, the aggressive behavior of the drug-resistant breast cancer cells.

### Targeting integrin α11 is an effective strategy to inhibit the growth and metastasis of drug-resistant breast cancer.

**Fig. 6:**
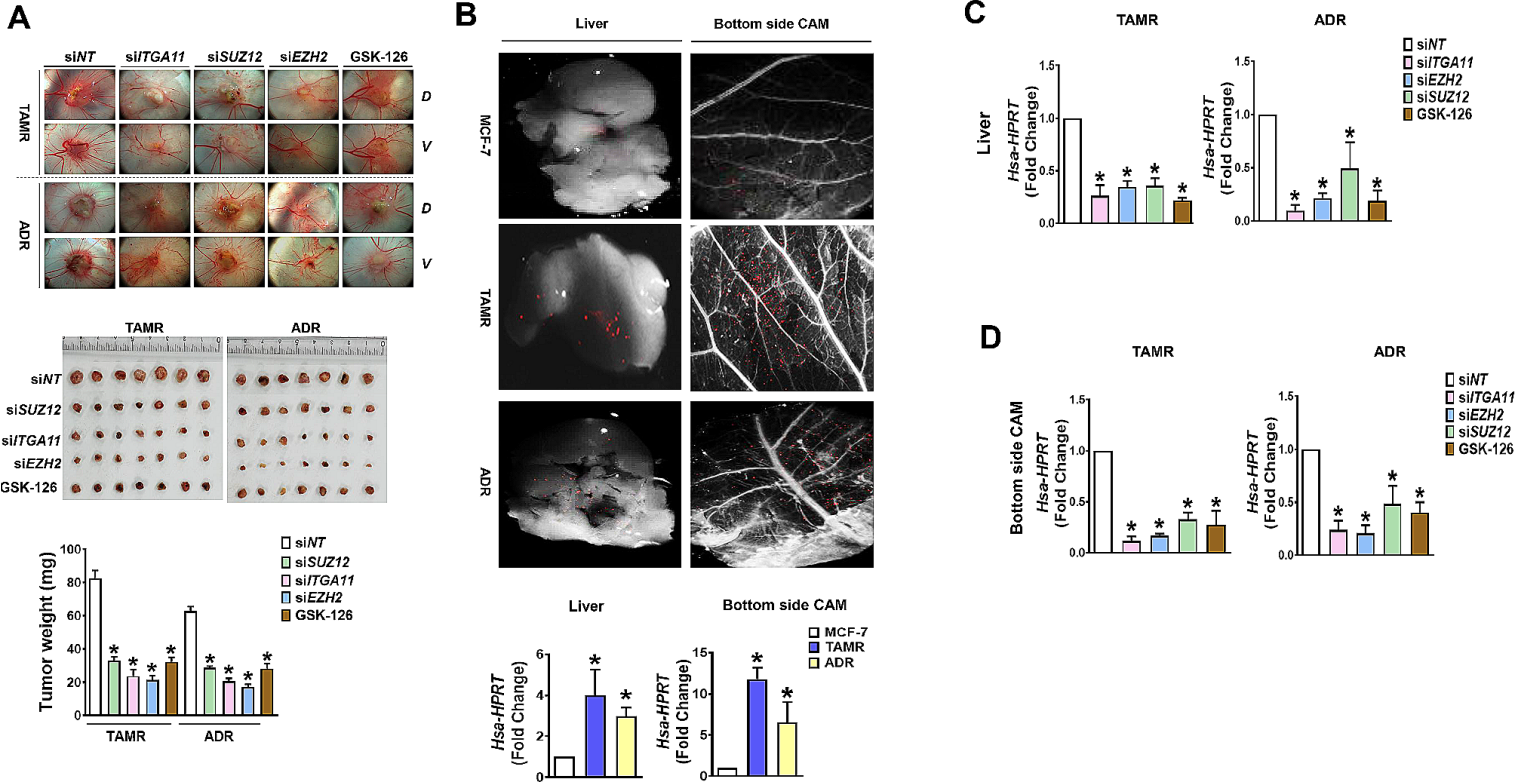
**Inhibition of EZH2-Integrin α11 suppresses growth and metastasis of CAM tumors with drug-resistant breast cancer** (A) Xenografted TAMR and ADR tumor growth and angiogenesis were inhibited by silencing of ITGA11, SUZ12, and EZH2, as well as EZH2 inhibitor GSK-126. The top panel shows photo image of tumors grown onto the CAM (dorsal, *D*) and angiogenesis on the ventral (*V*) sides of the cancer cell-inoculated membrane. The middle and bottom panels show the photographs of the tumor masses isolated from the xenografts and quantification of tumor weight, respectively. (B-D) Metastasis of tumor cells labeled with CMTPX, a cell-tracking red fluorescent dye, into liver and bottom side CAM tissue was inhibited by ITGA11/EZH2 axis inhibition. Compared to MCF-7 cells, TAMR and ADR cells exhibited more metastasis to the liver and opposite site (bottom) of the CAMs inoculated with cancer cells (B). The level of metastasis into liver and bottom CAM was quantified by qPCR for human housekeeping gene, *HPRT*. The effects of KD of ITGA11, SUZ12, and EZH2, and GSK126 treatment on metastasis to liver (C) and lower CAM (D) were quantified by human *HPRT* qPCR. *p < 0.05 compared to siNT-transfected group

The in vitro results suggest that overexpression of integrin α11 was the most critical event for the aggressive behavior of drug-resistant breast cancers. To validate the significance of EZH2-dependent integrin α11 in breast cancer progression, that is, whether integrin α11 over EZH2 is the right target to inhibit the growth and metastasis of drug-resistant breast cancers, two different xenograft tumor models were established, one in chick chorioallantoic membrane (CAM) and another in BALB/c nude mice. In the CAM tumor model that was implanted with parental and resistant cancer cells, the tumor growth observed at 5th day after implantation was significantly greater in the resistant cancer group (TAMR and ADR) than in parental MCF-7 cells, which corresponded to the tumor-induced angiogenesis measured by newly formed blood vessels (Supplementary Fig. S3). The tumor growth was significantly suppressed by KD of EZH2, SUZ12, or integrin α11 which was given to the cells at initial implantation (Fig. 6A). In addition, GSK-126, an EZH2 inhibitor, also suppressed the tumor growth less strongly than gene KD. In another set of CAM tumor models, cancer cells for implantation were pre-labeled with red fluorescent CMTPX dye to measure cancer metastasis. Compared to MCF-7 cells, siNT-treated TAMR and ADR cancer cells were more detected in the opposite (bottom) CAM and liver of the developing chicken embryo, which was detected under the stereomicroscope connected to fluorescence detector (Fig. 6B). The lower (bottom side) CAM and liver tissues were further analyzed to detect human housekeeping gene, hypoxanthine phosphoribosyl transferase (HPRT), using qPCR. The metastasis of the resistant cells to the liver (Fig. 6C) and bottom side CAM (Fig. 6D) was most significantly inhibited by KD of integrin α11, followed by EZH2 KD.

**Fig. 7:**
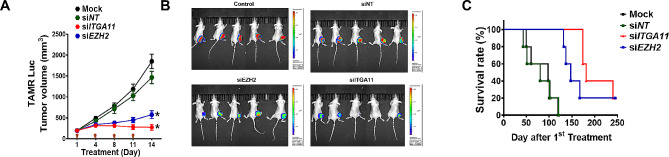
**Intratumoral administration of** ***ITGA11*** **siRNA prolongs survival of mice with drug-resistant breast cancer** (A) Tumor volume after intratumoral treatment with siRNA or GSK-126 at day 1, 4, 8, and 11, marked by arrows. Tumor volume was measured twice per week and significant differences in tumor volume compared to siNT are indicated at day 14. *p < 0.05 compared to Mock or siNT-transfected group. (B) Bioluminescent imaging by luciferase in TAMR xenografts at 3 days after the last treatment with siRNA or drug. D-luciferin (150 mg/kg) was injected via intraperitoneal injection in mice. At 10 min after D-luciferin injection, the converted D-luciferin was measured in the value of emitted photons. The tumor size in each group was shown by bioluminescent imaging by CCD camera. (C) Survival curves depicting the outcomes of the respective cohorts

In the mouse tumor model xenografted with TAMR cells, siRNAs were administered intratumorally into subcutaneous mouse xenografts twice a week for two weeks starting when the tumor size reached 200 mm^3^. After 2 times of drug administration, the tumor size in the siEZH2- or siITGA11-treated group was significantly smaller than the MOCK or siNT-treated group (Fig. 7A). At three days after the last siRNA drug administration, the tumor size in the si*EZH2*-treated group was significantly smaller but still growing, while the tumors in the si*ITGA11*-treated group stopped growing and showed a trend toward regression (Fig. 7A and B). The median survival of mice xenografted with TAMR cells was 98 days, and siNT treatment showed quite similar survival pattern (Fig. 7C). The vehicle- and siNT-treated control group experienced complete mortality with no surviving mice at day 120. In contrast, treatment with EZH2 and ITGA11 siRNA extended mean survival to 147 and 181 days, respectively. Notably, even after 240 days, 20% and 40% of the si*EZH2*- and si*ITGA11*-treated groups, respectively, survived.

## Discussion

Tamoxifen, a selective estrogen receptor (ER) modulator, serves as a cornerstone in adjuvant therapy, especially for premenopausal women, by directly antagonizing ER transcriptional activity. Despite its efficacy in reducing breast cancer recurrence and mortality rates, with reported reductions of 50% and 31%, respectively, approximately 20–30% of breast tumors exhibit resistance to tamoxifen therapy [48]. Tamoxifen resistance is multifactorial and may stem from various mechanisms, including alterations in ER signaling, crosstalk between ER and growth factor receptor networks, downregulation of ER expression, upregulation of specific growth factor receptors (GFRs), activation of the PI3K/AKT/mTOR pathway, PTEN inactivation, and induction of NFκB signaling [49]. Notably, integrin α6 [29] and breast cancer stem cells [50] have also been implicated in conferring tamoxifen resistance. Adriamycin, an anthracycline widely employed in contemporary therapeutics, is a key component of combination adjuvant breast cancer regimens, often administered alongside taxanes (paclitaxel and docetaxel) and fluorouracil/cyclophosphamide [51]. However, despite these treatment strategies, disease progression is observed in approximately 20–30% of patients with early-stage breast cancer following adjuvant therapy [52]. Anthracycline resistance mechanisms include alterations in drug transport proteins, augmentation of antioxidant defenses, dysregulation of apoptotic signaling pathways, and modulation of topoisomerase activity [53]. However, the involvement of integrins as a mechanism of anthracycline resistance has not been reported. The present study, for the first time, demonstrates integrin involvement in anthracycline resistance in breast cancer. Furthermore, the present study, using a comparative large-scale transcriptome analysis of TAMR and ADR cells, identified *ITGA11* as a master regulator that drives CSC expansion and drug resistance in breast cancer cells, independent of the type of anti-breast cancer drug.

Integrin α11 is normally expressed in mesenchymal cells and regulates the survival of mesenchymal stem cells [54]. The overexpression of α11 has been reported in fibrotic diseases of vital organs such as the lung, liver, and kidney [55], and desmoplastic tumor stroma development [56]. Although its role in epithelium-originated cancer and its potential as a diagnostic biomarker has been reported in non-small cell lung cancer patients [57], the present study is the first to report that integrin α11 may be a prognostic biomarker for drug-resistant breast cancer patients and an excellent therapeutic target for them, showing the results of cancer patient TCGA dataset analysis and *ITGA11* siRNA therapy in animal models of drug-resistant breast cancer.

Our transcriptome analysis also demonstrated that SUZ12 level was the most highly enhanced in CSCs of the drug-resistant breast cancer cells (TAMR and ADR). Alteration of SUZ12 by OV or KD correlated to the changes in CSC survival and expression of stemness-associated genes and *ITGA11*, and the effects were identical to those of EZH2 manipulation. In addition, the present study showed that SUZ12 gene KD did not alter EZH2 level, while EZH2 siRNA decreased SUZ12 gene expression, supporting the action mode of SUZ12, serving as a partner of EZH2, the catalytic subunit of Polycomb Repressive Complex 2 [58]. EZH2 normally is not expressed in healthy adult tissues but is only found in actively dividing cells, such as during fetal development [59]. In contrast to the action of EZH2 in developing embryonic cells, where it exerts transcriptional repression of genes involved in cell differentiation [60–63], overexpressed EZH2 in cancer cells induces growth-related gene expression in a PRC2-independent manner [62, 64]. Consistent with these reports, our study also demonstrated that EZH2 enhanced the expressions of stemness-associated TF genes, SOX2, OCT4, and Nanog, as well as SUZ12 in the drug-resistant breast cancer cells. Moreover, the present study demonstrated that EZH2, as a TF, induced gene expression of EZH2 itself and ITGA11.

The present study also showed that the upregulation of EZH2 and ITGA11 was associated with not only EZH2 but also other TFs, HIF1α, and GLI-1, to different degrees. ChIP-qPCR results showed that three TFs were bound to the EZH2 and ITGA11 genes in the order of HIF1α, EZH2, and GLI-1, but the KD effects of the three TFs on ITGA11 gene expression were the same. On the other hand, EZH2 gene expression was suppressed much more strongly by the EZH2 KD than by KD of HIF1α or GLI-1. Interestingly, our results showed HIF1α and GLI-1 expressions were also regulated by EZH2, which is consistent to the previous reports that the similar regulatory role of EZH2 in HIF1α and GLI-1 expressions has been shown in lung and pancreatic cancer cells [65, 66]. GLI transcription factors (GLI-1, GLI-2, GLI-3) are known to be activated by sonic hedgehog (Shh) binding to twelve transmembrane proteins patched 1 (Ptch), which subsequently removes the suppressive action of smoothened [67]. In breast cancer, Shh signaling components, including Ptch and GLI are overexpressed and correlate with estrogen-induced proliferation [68] and poor overall survival [69, 70]. In the present study, we demonstrated for the first time that GLI-1 is activated by integrin α11. Also, integrin α11-FAK-GLI-1 forms a key axis leading to a positive feedback circuit responsible for integrin α11 upregulation. Moreover, GLI-1 also upregulates EZH2 expression in cooperation with EZH2 themselves.

FAK, the key signaling component of integrin [71], is known to be activated through cytoplasmic tails of the integrin β1 subunits [72]. Although our co-IP results showed dimer formation of integrin α11 with β1, similar to previous reports [73], β1 levels in TAMR and ADR were not increased compared to the parental cells, making the imbalance in heterodimer formation in the resistant cells. The results indicate that α11 activates FAK via its cytoplasmic tail, which is consistent to the recent findings [74]. In addition to the previous report that integrin α6β1-activated FAK induces GLI-1 expression in triple-negative breast cancer [28], our present results with ITGA11 KD demonstrated that integrin α11 induced activation (nuclear translocation) of GLI-1, but not HIF1α and EZH2. Moreover, the findings that β1 KD in the TAMR and ADR did not recover sensitivity to the anticancer drugs further supported that overexpressed α11, but not β1, was the master regulator for activating intracellular signaling and inducing chemoresistance.

**Fig. 8:**
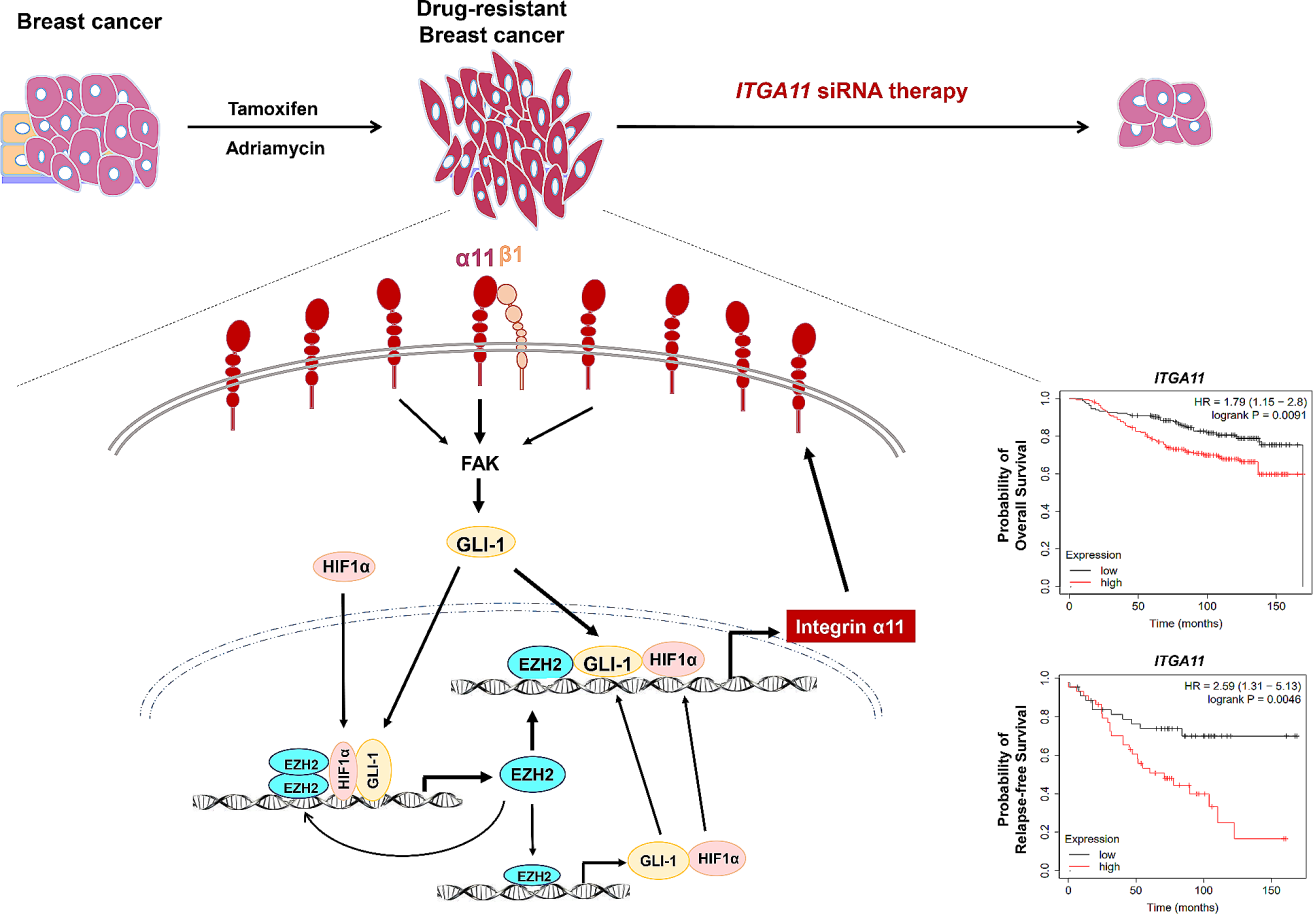
Schematic diagram unraveling the regulatory mechanism and clinical implications of integrin α11 upregulation in drug-resistant breast cancer

The axis of integrin α11-FAK-GLI-1/EZH2, formed in drug-resistant breast cancer cells, represents a shared positive feedback loop in both TAMR and ADR cells, which sustains the overexpression of EZH2 and ITGA11, thereby inducing drug resistance and CSC expansion (Fig. 8). The pivotal roles of integrin α11, and EZH2, which regulates integrin α11 expression along with GLI-1, suggest them as potential targets for inhibiting drug-resistant breast cancer. However, the antitumor effects of siRNA in a mouse xenograft tumor model demonstrated that ITGA11 siRNA treatment outperformed EZH2 siRNA treatment, indicating the superior efficacy of ITGA11 siRNA in suppressing tumor growth.

## Conclusions

In summary, our findings elucidate the regulatory mechanism underlying the upregulation of integrin α11 in drug-resistant breast cancer cells (Fig. 8). Integrin α11 which was consistently up-regulated by cooperative interactions of HIF1α, GLI-1, and EZH2 in a positive feedback circuit, resulted in drug resistance, CSC increase and EMT in breast cancer cells. Both EZH2 and integrin α11 were found to be strong prognostic factors of relapse-free and overall survival of patients with breast cancer, but integrin α11 was a more effective therapeutic target than EZH2 for the treatment of drug-resistant breast cancer.

### Electronic supplementary material

Below is the link to the electronic supplementary material.

Supplementary Material 1

Supplementary Material 2

## Data Availability

Nanostringn Ncounter Pancancer Pathway array data generated in this study are available upon request from the corresponding author (jakim@yu.ac.kr).

## Abbreviations

ADR: Adriamycin-resistant MCF-7 cells
CSCs: ImplementatiCancer stem cells
DEG: Differentially expressed genes
EMT: Epithelial-mesenchymal transition
ER+: Estrogen receptor-positive
FAK: focal adhesion kinase
GEO: Gene Expression Omnibus
GSEA: Gene set enrichment analysis
HER2+: Human epidermal growth factor receptor 2-positive
HPRT: Hypoxanthine phosphoribosyl transferase
OS: Overall survival
RFS: Relapse-free survival
TAMR: Tamoxifen-resistant MCF-7 cells
TCGA: The Cancer Genome Atlas
TFs: Transcription factors
TNBC: Triple-negative breast cancer

## References

[1] Arnold M, Morgan E, Rumgay H, Mafra A, Singh D, Laversanne M (2022). Current and future burden of breast cancer: global statistics for 2020 and 2040. Breast.

[2] Wilkinson L, Gathani T (2022). Understanding breast cancer as a global health concern. Brit J Radiol.

[3] Gautam J, Banskota S, Regmi SC, Ahn S, Jeon YH, Jeong H (2016). Tryptophan hydroxylase 1 and 5-HT7 receptor preferentially expressed in triple-negative breast cancer promote cancer progression through autocrine serotonin signaling. Mol Cancer.

[4] Goldberg J, Pastorello RG, Vallius T, Davis J, Cui YX, Agudo J (2021). The immunology of hormone receptor positive breast cancer. Front Immunol.

[5] Hanker AB, Sudhan DR, Arteaga CL (2020). Overcoming endocrine resistance in breast cancer. Cancer Cell.

[6] Gote V, Nookala AR, Bolla PK, Pal D (2021). Drug resistance in metastatic breast cancer: tumor targeted nanomedicine to the rescue. Int J Mol Sci.

[7] Hernando C, Ortega-Morillo B, Tapia M, Moragón S, Martínez MT, Eroles P (2021). Oral selective estrogen receptor degraders (SERDs) as a novel breast cancer therapy: present and future from a clinical perspective. Int J Mol Sci.

[8] Patel HK, Bihani T (2018). Selective estrogen receptor modulators (SERMs) and selective estrogen receptor degraders (SERDs) in cancer treatment. Pharmacol Ther.

[9] Group EBCTC (2005). Effects of chemotherapy and hormonal therapy for early breast cancer on recurrence and 15-year survival: an overview of the randomised trials. Lancet.

[10] Najafi S, Sadeghi M, Shajari MR, Abasvandi F, Mohebi K (2018). The comparison of anthracycline-based and non-anthracycline-based regimens in adjuvant chemotherapy of HER2-positive non-metastatic breast cancers. Contemp Oncol (Pozn).

[11] Hurvitz SA, McAndrew NP, Bardia A, Press MF, Pegram M, Crown JP (2021). A careful reassessment of anthracycline use in curable breast cancer. NPJ Breast Cancer.

[12] Rampurwala MM, Rocque GB, Burkard ME (2014). Update on adjuvant chemotherapy for early breast cancer. Breast Cancer: Basic Clin Res.

[13] Hosford SR, Miller TW. Clinical potential of novel therapeutic targets in breast cancer: CDK4/6, Src, JAK/STAT, PARP, HDAC, and PI3K/AKT/mTOR pathways. Pharmgenomics Pers Med. 2014: 203–15. 10.2147/PGPM.S52762 PMC4157397 25206307

[14] Kumar R, Mandal M, Lipton A, Harvey H, Thompson CB (1996). Overexpression of HER2 modulates bcl-2, bcl-XL, and tamoxifen-induced apoptosis in human MCF-7 breast cancer cells. Clin Cancer Res.

[15] Rimawi MF, De Angelis C, Schiff R (2015). Resistance to anti-HER2 therapies in breast cancer. Am Soc Clin Oncol Educ Book.

[16] Wu X, Yang H, Yu X, Qin J-J (2022). Drug-resistant HER2-positive breast cancer: molecular mechanisms and overcoming strategies. Front Pharmacol.

[17] Rexer BN, Arteaga CL. Intrinsic and acquired resistance to HER2-targeted therapies in HER2 gene-amplified breast cancer: mechanisms and clinical implications. Crit Rev Oncog. 2012, 17(1). 10.1615/critrevoncog.v17.i1.20 PMC3394454 22471661

[18] Cazet AS, Hui MN, Elsworth BL, Wu SZ, Roden D, Chan C-L (2018). Targeting stromal remodeling and cancer stem cell plasticity overcomes chemoresistance in triple-negative breast cancer. Nat Commun.

[19] Barbato L, Bocchetti M, Di Biase A, Regad T (2019). Cancer stem cells and targeting strategies. Cells.

[20] Seguin L, Desgrosellier JS, Weis SM, Cheresh DA (2015). Integrins and cancer: regulators of cancer stemness, metastasis, and drug resistance. Trends Cell Biol.

[21] Cooper J, Giancotti FG (2019). Integrin signaling in cancer: mechanotransduction, stemness, epithelial plasticity, and therapeutic resistance. Cancer Cell.

[22] Maugeri-Saccà M, Vigneri P, De Maria R (2011). Cancer stem cells and chemosensitivity. Clin Cancer Res.

[23] Kotiyal S, Bhattacharya S (2014). Breast cancer stem cells, EMT and therapeutic targets. Biochem Biophys Res Commun.

[24] Giancotti FG, Tarone G (2003). Positional control of cell fate through joint integrin/receptor protein kinase signaling. Annu Rev Cell Dev Biol.

[25] Yousefi H, Vatanmakanian M, Mahdiannasser M, Mashouri L, Alahari NV, Monjezi MR (2021). Understanding the role of integrins in breast cancer invasion, metastasis, angiogenesis, and drug resistance. Oncogene.

[26] Zutter MM. Integrin-mediated adhesion: tipping the balance between chemosensitivity and chemoresistance. Adv Exp Med Biol. 2007: 87–100. 10.1007/978-0-387-74039-3_6 17993234

[27] Xiong J, Yan L, Zou C, Wang K, Chen M, Xu B (2021). Integrins regulate stemness in solid tumor: an emerging therapeutic target. J Hematol Oncol.

[28] Goel HL, Pursell B, Chang C, Shaw LM, Mao J, Simin K (2013). GLI1 regulates a novel neuropilin-2/α6β1 integrin based autocrine pathway that contributes to breast cancer initiation. EMBO Mol Med.

[29] Campbell PS, Mavingire N, Khan S, Rowland LK, Wooten JV, Opoku-Agyeman A (2019). AhR ligand aminoflavone suppresses α6‐integrin–src–akt signaling to attenuate tamoxifen resistance in breast cancer cells. J Cell Physiol.

[30] Adorno-Cruz V, Hoffmann AD, Liu X, Wray B, Keri RA, Liu H. ITGA2 is a target of miR-206 promoting cancer stemness and lung metastasis through enhanced ACLY and CCND1 expression in triple negative breast cancer. bioRxiv. 2019: 583062.

[31] Wang Z, Li Y, Xiao Y, Lin HP, Yang P, Humphries B (2019). Integrin α9 depletion promotes β-catenin degradation to suppress triple‐negative breast cancer tumor growth and metastasis. Int J Cancer.

[32] Barnawi R, Al-Khaldi S, Colak D, Tulbah A, Al‐Tweigeri T, Fallatah M (2019). β1 integrin is essential for fascin‐mediated breast cancer stem cell function and disease progression. Int J Cancer.

[33] Zhang Y, Zhang Q, Cao Z, Huang Y, Cheng S, Pang D (2018). HOXD3 plays a critical role in breast cancer stemness and drug resistance. Cell Physiol Biochem.

[34] Taddei I, Deugnier MA, Faraldo MM, Petit V, Bouvard D, Medina D (2008). β1 integrin deletion from the basal compartment of the mammary epithelium affects stem cells. Nat Cell Biol.

[35] Zhang L, Qu J, Qi Y, Duan Y, Huang Y-W, Zhou Z (2022). EZH2 engages TGFβ signaling to promote breast cancer bone metastasis via integrin β1-FAK activation. Nat Commun.

[36] Dahal S, Chaudhary P, Kim J-A. Induction of promyelocytic leukemia zinc finger protein by miR-200c-3p restores sensitivity to anti-androgen therapy in androgen-refractory prostate cancer and inhibits the cancer progression via down-regulation of integrin α3β4. Cell Oncol. 2023: 1–14. 10.1007/s13402-023-00803-y PMC12974746 36995683

[37] Mi H, Ebert D, Muruganujan A, Mills C, Albou L-P, Mushayamaha T (2021). PANTHER version 16: a revised family classification, tree-based classification tool, enhancer regions and extensive API. Nucleic Acids Res.

[38] Pinto JP, Kalathur RK, Oliveira DV, Barata T, Machado RS, Machado S (2015). StemChecker: a web-based tool to discover and explore stemness signatures in gene sets. Nucleic Acids Res.

[39] Vistica DT, Skehan P, Scudiero D, Monks A, Pittman A, Boyd MR (1991). Tetrazolium-based assays for Cellular viability: a critical examination of selected parameters affecting Formazan production. Cancer Res.

[40] Zhang Y, Wang M, Zhang X, Jiang Z, Zhang Y, Fu X et al. Helicid improves lipopolysaccharide-induced apoptosis of C6 cells by regulating SH2D5 DNA methylation via the CytC/Caspase9/Caspase3 signaling pathway. Contrast Media Mol I. 2022, 2022. 10.1155/2022/9242827 PMC8820944 35173561

[41] Romaine A, Melleby AO, Alam J, Lobert VH, Lu N, Lockwood FE (2022). Integrin α11β1 and syndecan-4 dual receptor ablation attenuate cardiac hypertrophy in the pressure overloaded heart. Am J Physiol Heart Circ Physiol.

[42] Shen B, Vardy K, Hughes P, Tasdogan A, Zhao Z, Yue R (2019). Integrin alpha11 is an osteolectin receptor and is required for the maintenance of adult skeletal bone mass. Elife.

[43] Nutt SL, Keenan C, Chopin M, Allan RS (2020). EZH2 function in immune cell development. Biol Chem.

[44] Cyrus S, Burkardt D, Weaver DD, Gibson WT. PRC2-complex related dysfunction in overgrowth syndromes: a review of EZH2, EED, and SUZ12 and their syndromic phenotypes. Am J Med Genet C Semin Med Genet. 2019. p. 519–31. 10.1002/ajmg.c.31754 31724824

[45] Pastushenko I, Blanpain C (2019). EMT transition states during tumor progression and metastasis. Trends Cell Biol.

[46] Najafi M, Farhood B, Mortezaee K (2019). Cancer stem cells (CSCs) in cancer progression and therapy. J Cell Physiol.

[47] Celià-Terrassa T, Jolly MK (2020). Cancer stem cells and epithelial-to-mesenchymal transition in cancer metastasis. Cold Spring Harb Perspect Med.

[48] Ali S, Mondal N, Choudhry H, Rasool M, Pushparaj PN, Khan MA et al. Current management strategies in breast Cancer by targeting key altered molecular players. Front Oncol. 2016, 6. 10.3389/fonc.2016.00045 PMC4771739 26973813

[49] Hosford SR, Miller TW (2014). Clinical potential of novel therapeutic targets in breast cancer: CDK4/6, Src, JAK/STAT, PARP, HDAC, and PI3K/AKT/mTOR pathways. Pharmgenomics Pers Med.

[50] Droog M, Beelen K, Linn S, Zwart W (2013). Tamoxifen resistance: from bench to bedside. Eur J Pharmacol.

[51] Hernandez-Aya LF, Gonzalez-Angulo AM (2013). Adjuvant systemic therapies in breast Cancer. Surg Clin North Am.

[52] Berman AT, Thukral AD, Hwang W-T, Solin LJ, Vapiwala N (2013). Incidence and patterns of distant metastases for patients with early-stage breast Cancer after breast conservation treatment. Clin Breast Cancer.

[53] Chien AJ, Moasser MM (2008). Cellular mechanisms of Resistance to anthracyclines and taxanes in Cancer: intrinsic and acquired. Semin Oncol.

[54] Popov C, Radic T, Haasters F, Prall W, Aszodi A, Gullberg D (2011). Integrins α2β1 and α11β1 regulate the survival of mesenchymal stem cells on collagen I. Cell Death Dis.

[55] Bansal R, Nakagawa S, Yazdani S, Van Baarlen J, Venkatesh A, Koh AP (2017). Integrin alpha 11 in the regulation of the myofibroblast phenotype: implications for fibrotic diseases. Exp Mol Med.

[56] Zeltz C, Alam J, Liu H, Erusappan PM, Hoschuetzky H, Molven A (2019). α11β1 integrin is induced in a subset of cancer-associated fibroblasts in desmoplastic tumor stroma and mediates in vitro cell migration. Cancers.

[57] Wu P, Wang Y, Wu Y, Jia Z, Song Y, Liang N (2019). Expression and prognostic analyses of ITGA11, ITGB4 and ITGB8 in human non-small cell lung cancer. PeerJ.

[58] Pasini D, Bracken AP, Jensen MR, Denchi EL, Helin K (2004). Suz12 is essential for mouse development and for EZH2 histone methyltransferase activity. EMBO J.

[59] Konze KD, Ma A, Li F, Barsyte-Lovejoy D, Parton T, MacNevin CJ (2013). An orally bioavailable chemical probe of the lysine methyltransferases EZH2 and EZH1. ACS Chem Biol.

[60] O’Meara MM, Simon JA (2012). Inner workings and regulatory inputs that control polycomb repressive complex 2. Chromosoma.

[61] Ding X, Wang X, Sontag S, Qin J, Wanek P, Lin Q (2014). The polycomb protein Ezh2 impacts on induced pluripotent stem cell generation. Stem Cells Dev.

[62] Tan J-z, Yan Y, Wang X-x, Jiang Y, Xu HE (2014). EZH2: biology, disease, and structure-based drug discovery. Acta Pharmacol Sin.

[63] Lund K, Adams P, Copland M (2014). EZH2 in normal and malignant hematopoiesis. Leukemia.

[64] Chaudhary P, Guragain D, Chang J-H, Kim J-A (2021). TPH1 and 5-HT7 receptor overexpression leading to Gemcitabine-Resistance requires non-canonical permissive action of EZH2 in pancreatic ductal adenocarcinoma. Cancers.

[65] Zhao Y, Wang X-X, Wu W, Long H, Huang J, Wang Z (2019). EZH2 regulates PD-L1 expression via HIF-1α in non-small cell lung cancer cells. Biochem Biophys Res Commun.

[66] Li H, Wang H, Cui Y, Jiang W, Zhan H, Feng L (2023). EZH2 regulates pancreatic cancer cells through E2F1, GLI1, CDK3, and Mcm4. Hereditas.

[67] Katano M (2005). Hedgehog signaling pathway as a therapeutic target in breast cancer. Cancer Lett.

[68] Koga K, Nakamura M, Nakashima H, Akiyoshi T, Kubo M, Sato N (2008). Novel link between estrogen receptor α and hedgehog pathway in breast Cancer. Anticancer Res.

[69] Im S, Choi HJ, Yoo C, Jung J-H, Jeon Y-W, Suh YJ (2013). Hedgehog related protein expression in breast Cancer: Gli-2 is Associated with poor overall survival. Korean J Pathol.

[70] Wang B, Yu T, Hu Y, Xiang M, Peng H, Lin Y et al. Prognostic role of Gli1 expression in breast cancer: a meta-analysis. Oncotarget 2017, 8(46). 10.18632/oncotarget.19080 PMC5655264 29113369

[71] Glénisson M, Vacher S, Callens C, Susini A, Cizeron-Clairac G, Le Scodan R (2012). Identification of new candidate therapeutic target genes in triple-negative breast cancer. Genes Cancer.

[72] Shibue T, Weinberg RA (2009). Integrin β1-focal adhesion kinase signaling directs the proliferation of metastatic cancer cells disseminated in the lungs. Proc Natl Acad Sci U S A.

[73] Popova S, Lundgren-Åkerlund E, Wiig H, Gullberg D (2007). Physiology and pathology of collagen receptors. Acta Physiol.

[74] Erusappan P, Alam J, Lu N, Zeltz C, Gullberg D (2019). Integrin α11 cytoplasmic tail is required for FAK activation to initiate 3D cell invasion and ERK-mediated cell proliferation. Sci Rep.

