# Supplementary material for: siRNA treatment targeting integrin α11 overexpressed via EZH2-driven axis inhibits drug-resistant breast cancer progression
Source: Breast Cancer Res. 2024 Apr 25;26:72. doi: 10.1186/s13058-024-01827-4 (PMC11046805; doi:10.1186/s13058-024-01827-4)
Supplement: Supplementary file 1 — Supplementary Material 1 [file 13058_2024_1827_MOESM1_ESM.pdf]

siRNA treatment targeting integrin α11 overexpressed via EZH2-driven axis inhibits drug-resistant breast cancer progression

Prakash Chaudhary[1], Kiran Yadav, Ho Jin Lee[2], Keon Wook Kang[2], Jongseo Mo, Jung-Ae Kim[1]*

[1]College of Pharmacy, Yeungnam University, Gyeongsan 38541, Republic of Korea

[2]College of Pharmacy and Research Institute of Pharmaceutical Sciences, Seoul National University, Seoul 08826, Republic of Korea

*Correspondence to: jakim@ynu.ac.kr

Jung-Ae Kim, Ph.D., College of Pharmacy,

Yeungnam University, Gyeongsan 38541, Republic of Korea;

Phone: +82-53-810-2816; Fax: +82-53-810-4654; E-mail: jakim@yu.ac.kr

Supplementary Fig. S1

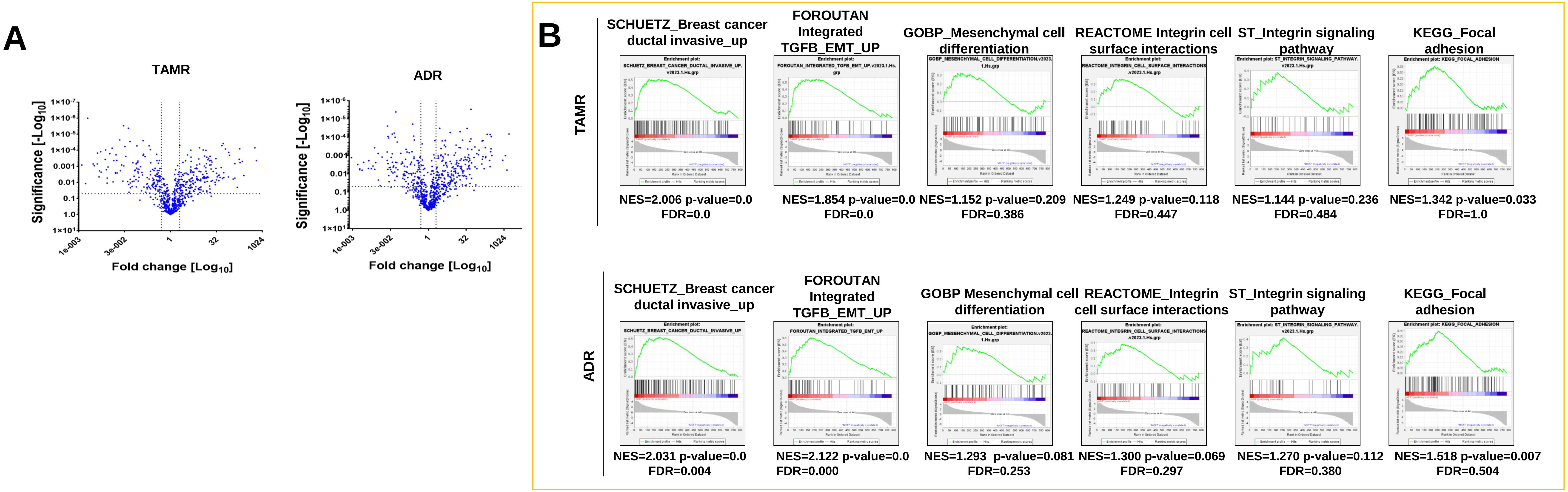

Supplementary Figure S1. Transcriptome analysis of drug-resistant cells relative to MCF-7.

(A) Scatter plot illustrating the differential gene expression values in drug-resistant cells compared to MCF-7 cells. (B) Enrichment plots depicting various datasets for several epithelial-mesenchymal transition (EMT)-related pathways.

Supplementary Fig. S2

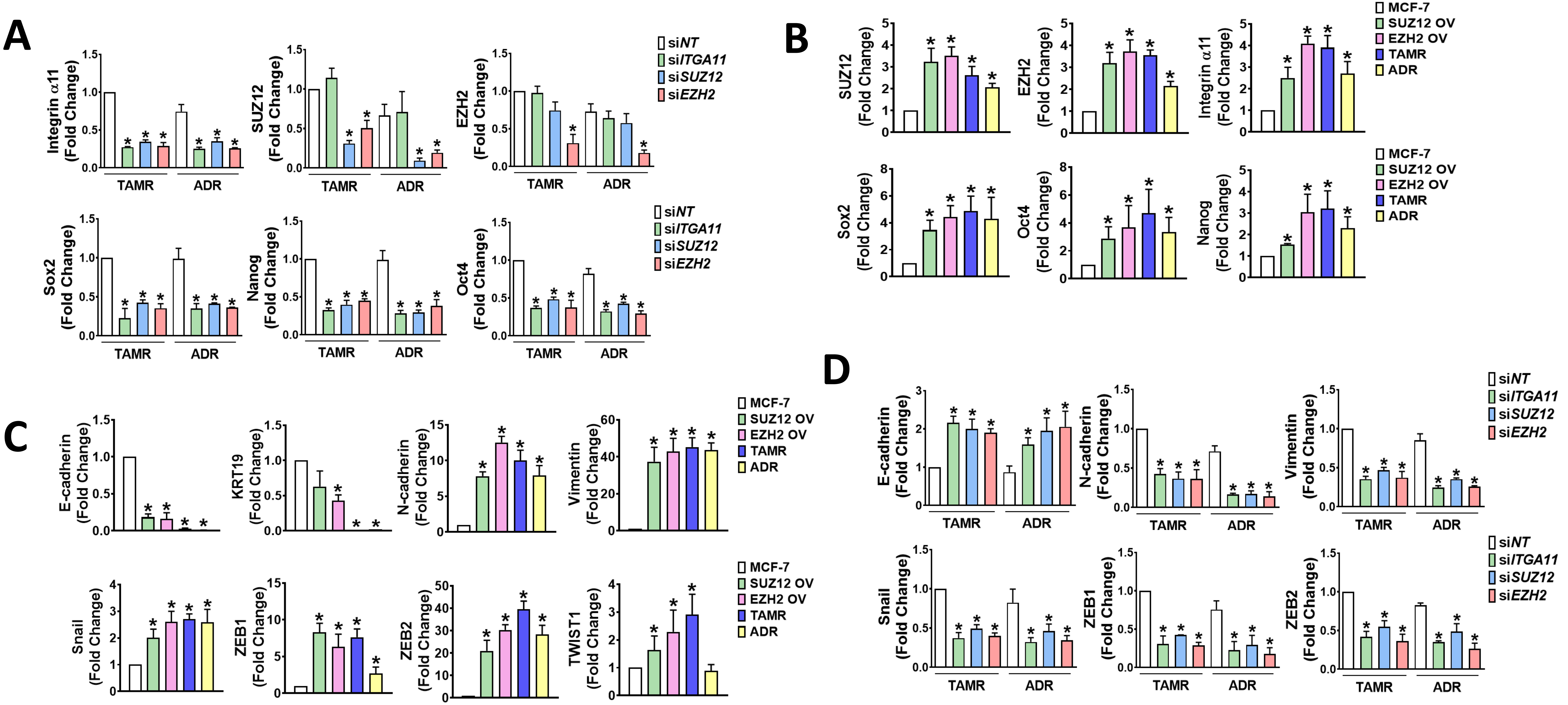

Supplementary Figure 2. Impact of ITGA11, SUZ12, and EZH2 modulation on protein expression

(A) Graph illustrating the effect of ITGA11, SUZ12, or EZH2 silencing on the expression of indicated proteins. (B) Graph depicting the effect of ITGA11, SUZ12, or EZH2 overexpression on the expression of indicated proteins. (C) Comparative analysis demonstrating the influence of SUZ12 or EZH2 overexpression in resistant cell lines, as compared to MCF-7 cells, on the expression of epithelial cell marker proteins (E-Cadherin, Keratin19) and mesenchymal cell marker proteins (N-cadherin, Vimentin, Snail, ZEB1, ZEB2, TWIST1). (D) Comparative analysis revealing the impact of ITGA11, SUZ12, or EZH2 silencing in resistant cells on the expression of epithelial cell marker proteins (E-cadherin, Keratin19) and mesenchymal cell marker proteins (N-cadherin, Vimentin, Snail, ZEB1, ZEB2, TWIST1). Fold change values are calculated from three independent experiments and displayed in the bar graph.

Supplementary Fig. S3

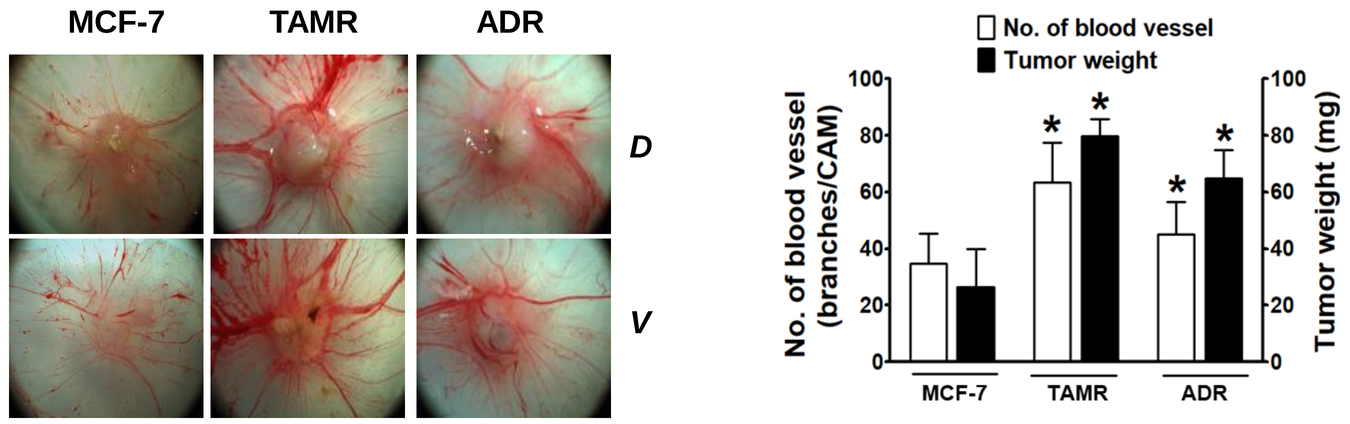

Supplementary Figure 3. Tumor growth and tumor-induced angiogenesis analysis

(Left) Photo image of the lower side of the chorioallantoic membrane (CAM) following xenograft, with D and V indicating the dorsal and ventral sides of the cancer cell-inoculated membrane. (Right) Harvested tumor mass subjected to further analysis, including measurement of its weight (black filled) and quantification of the number of newly formed vessel branches (white filled).
