# Supplementary material for: siRNA treatment targeting integrin α11 overexpressed via EZH2-driven axis inhibits drug-resistant breast cancer progression
Source: Breast Cancer Res. 2024 Apr 25;26:72. doi: 10.1186/s13058-024-01827-4 (PMC11046805; doi:10.1186/s13058-024-01827-4)

Full Uncropped Western Blots

for

siRNA treatment targeting integrin α11 overexpressed via EZH2-driven axis inhibits drug-resistant breast cancer progression

Prakash Chaudhary[1], Kiran Yadav, Ho Jin Lee[2], Keon Wook Kang[2], Jongseo Mo, Jung-Ae Kim[1]*

[1]College of Pharmacy, Yeungnam University, Gyeongsan 38541, Republic of Korea

[2]College of Pharmacy and Research Institute of Pharmaceutical Sciences, Seoul National University, Seoul 08826, Republic of Korea

*Correspondence to: jakim@ynu.ac.kr

Jung-Ae Kim, Ph.D., College of Pharmacy,

Yeungnam University, Gyeongsan 38541, Republic of Korea;

Phone: +82-53-810-2816; Fax: +82-53-810-4654; E-mail: jakim@yu.ac.kr

Fig. 1H

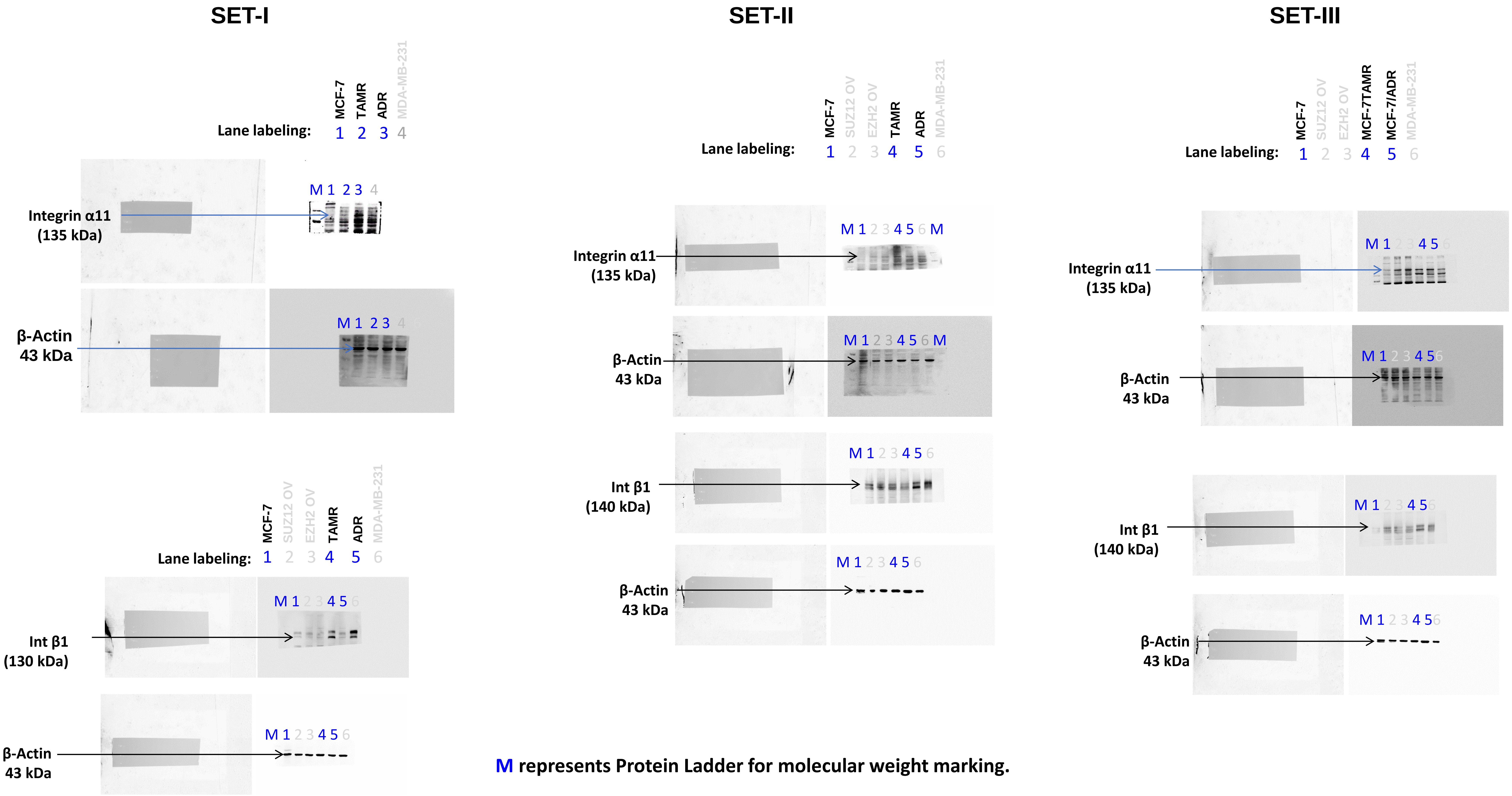

M represents Protein Ladder for molecular weight marking.

Fig. 1I

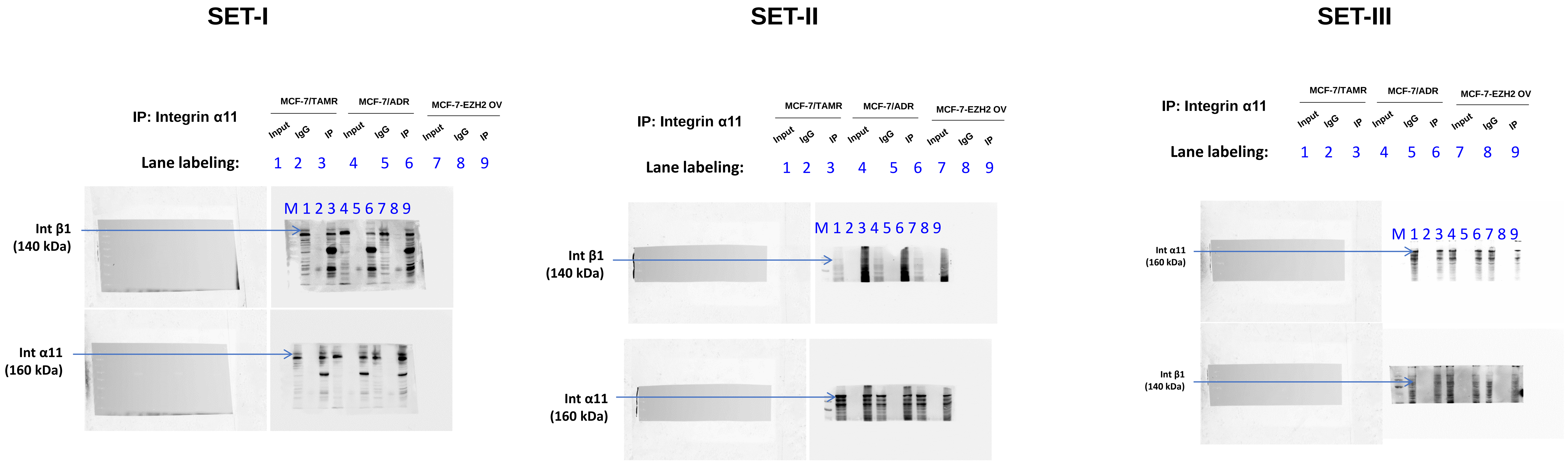
SET-I
IP: Integrin α11
MCF-7/TAMR
MCF-7/ADR
MCF-7-EZH2 OV
Input IgG IP Input IgG IP Input IgG IP
Lane labeling: 1 2 3 4 5 6 7 8 9
M 1 2 3 4 5 6 7 8 9
Int β1 (140 kDa)
Int α11 (160 kDa)
SET-II
IP: Integrin α11
MCF-7/TAMR
MCF-7/ADR
MCF-7-EZH2 OV
Input IgG IP Input IgG IP Input IgG IP
Lane labeling: 1 2 3 4 5 6 7 8 9
M 1 2 3 4 5 6 7 8 9
Int β1 (140 kDa)
Int α11 (160 kDa)
SET-III
IP: Integrin α11
MCF-7/TAMR
MCF-7/ADR
MCF-7-EZH2 OV
Input IgG IP Input IgG IP Input IgG IP
Lane labeling: 1 2 3 4 5 6 7 8 9
M 1 2 3 4 5 6 7 8 9
Int α11 (160 kDa)
Int β1 (140 kDa)

Fig. 2F

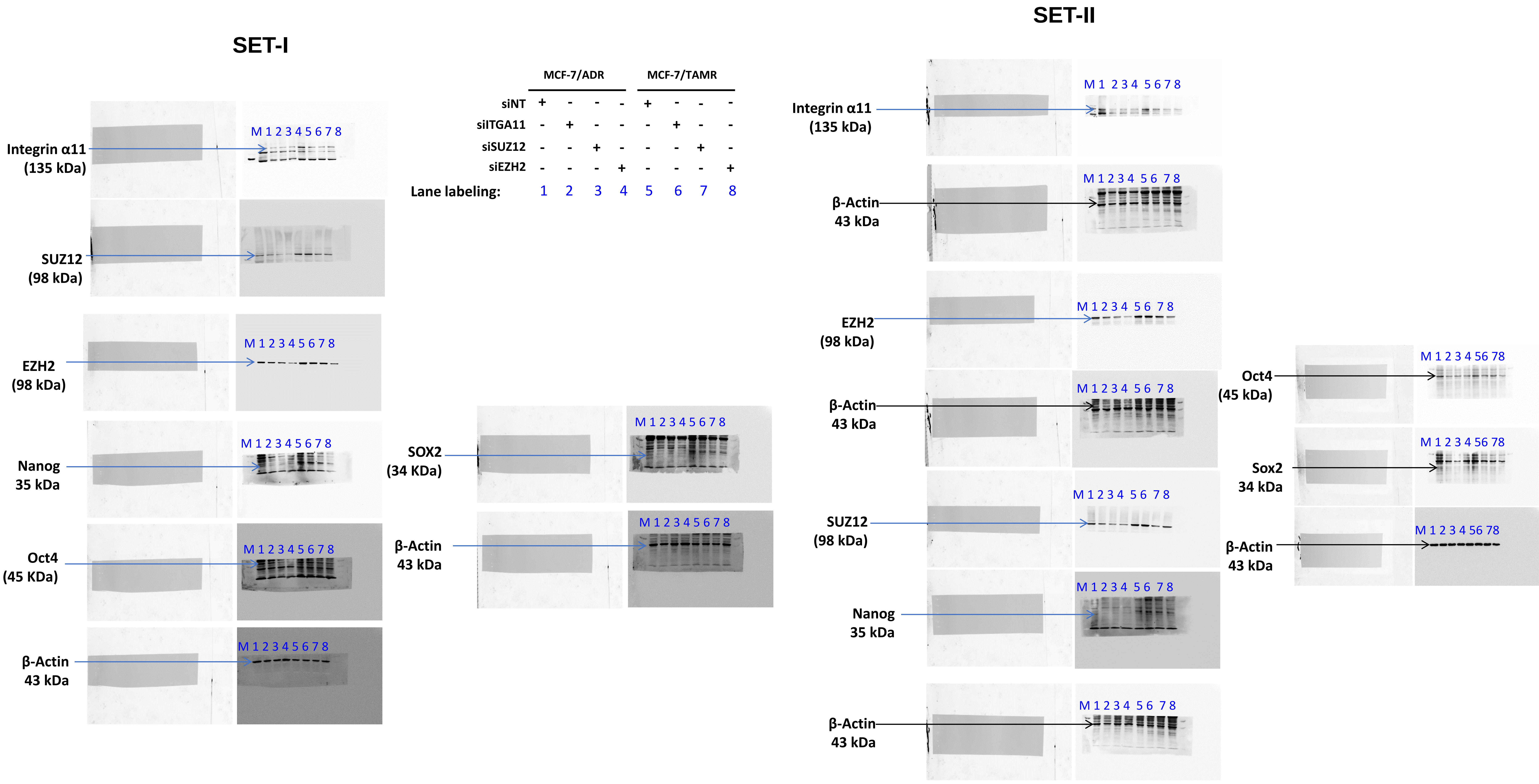
SET-I
SET-II
MCF-7/ADR
MCF-7/TAMR
siNT + - - - + - - -
siITGA11 - + - - - + - -
siSUZ12 - - + - - - + -
siEZH2 - - - + - - - +
Lane labeling: 1 2 3 4 5 6 7 8
M 1 2 3 4 5 6 7 8
Integrin α11 (135 kDa)
SUZ12 (98 kDa)
EZH2 (98 kDa)
Nanog 35 kDa
Oct4 (45 KDa)
β-Actin 43 kDa
SOX2 (34 KDa)
Oct4 (45 kDa)
Sox2 34 kDa

Fig. 2F

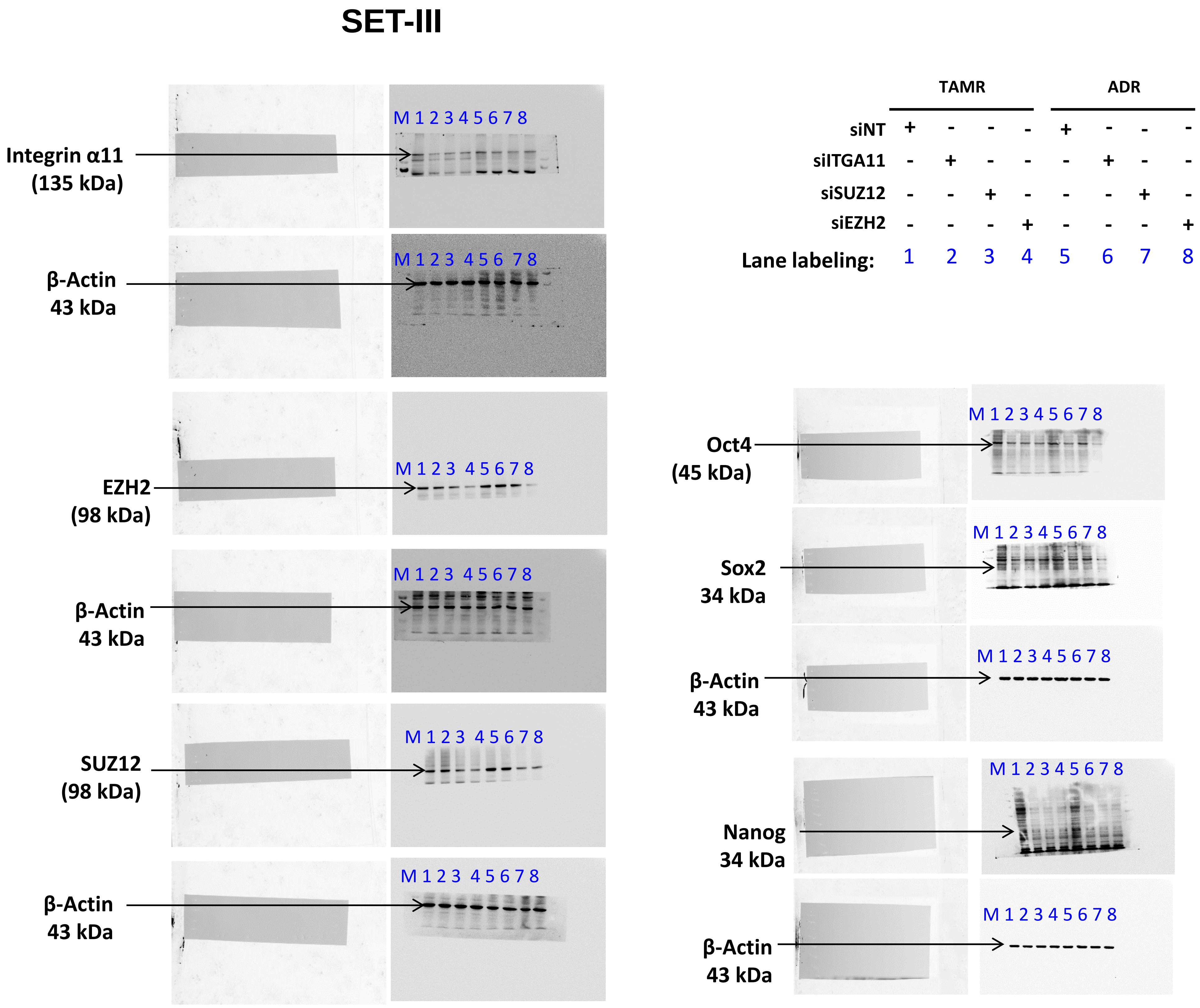
SET-III
TAMR
ADR
siNT + - - - + - - -
siITGA11 - + - - - + - -
siSUZ12 - - + - - - + -
siEZH2 - - - + - - - +
Lane labeling: 1 2 3 4 5 6 7 8
M 1 2 3 4 5 6 7 8
Integrin α11 (135 kDa)
β-Actin 43 kDa
EZH2 (98 kDa)
SUZ12 (98 kDa)
Oct4 (45 kDa)
Sox2 34 kDa
Nanog 34 kDa

Fig. 3B

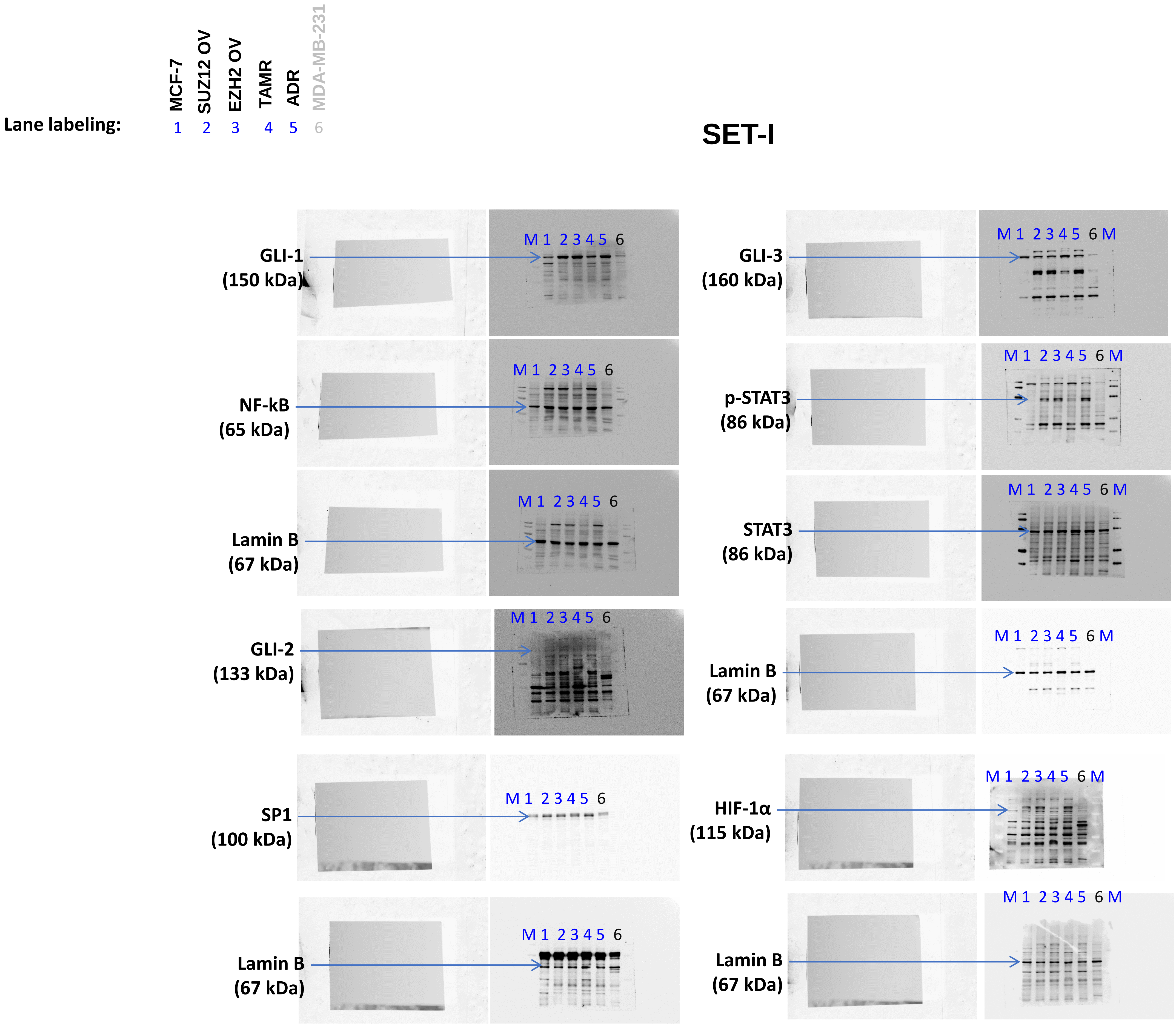
Lane labeling:
MCF-7
SUZ12 OV
EZH2 OV
TAMR
ADR
MDA-MB-231
1 2 3 4 5 6
SET-I
M 1 2 3 4 5 6
GLI-1
(150 kDa)
M 1 2 3 4 5 6 M
GLI-3
(160 kDa)
M 1 2 3 4 5 6
NF-kB
(65 kDa)
M 1 2 3 4 5 6 M
p-STAT3
(86 kDa)
M 1 2 3 4 5 6
Lamin B
(67 kDa)
M 1 2 3 4 5 6 M
STAT3
(86 kDa)
M 1 2 3 4 5 6
GLI-2
(133 kDa)
M 1 2 3 4 5 6 M
Lamin B
(67 kDa)
M 1 2 3 4 5 6
SP1
(100 kDa)
M 1 2 3 4 5 6 M
HIF-1α
(115 kDa)
M 1 2 3 4 5 6
Lamin B
(67 kDa)
M 1 2 3 4 5 6 M
Lamin B
(67 kDa)

Fig. 3B

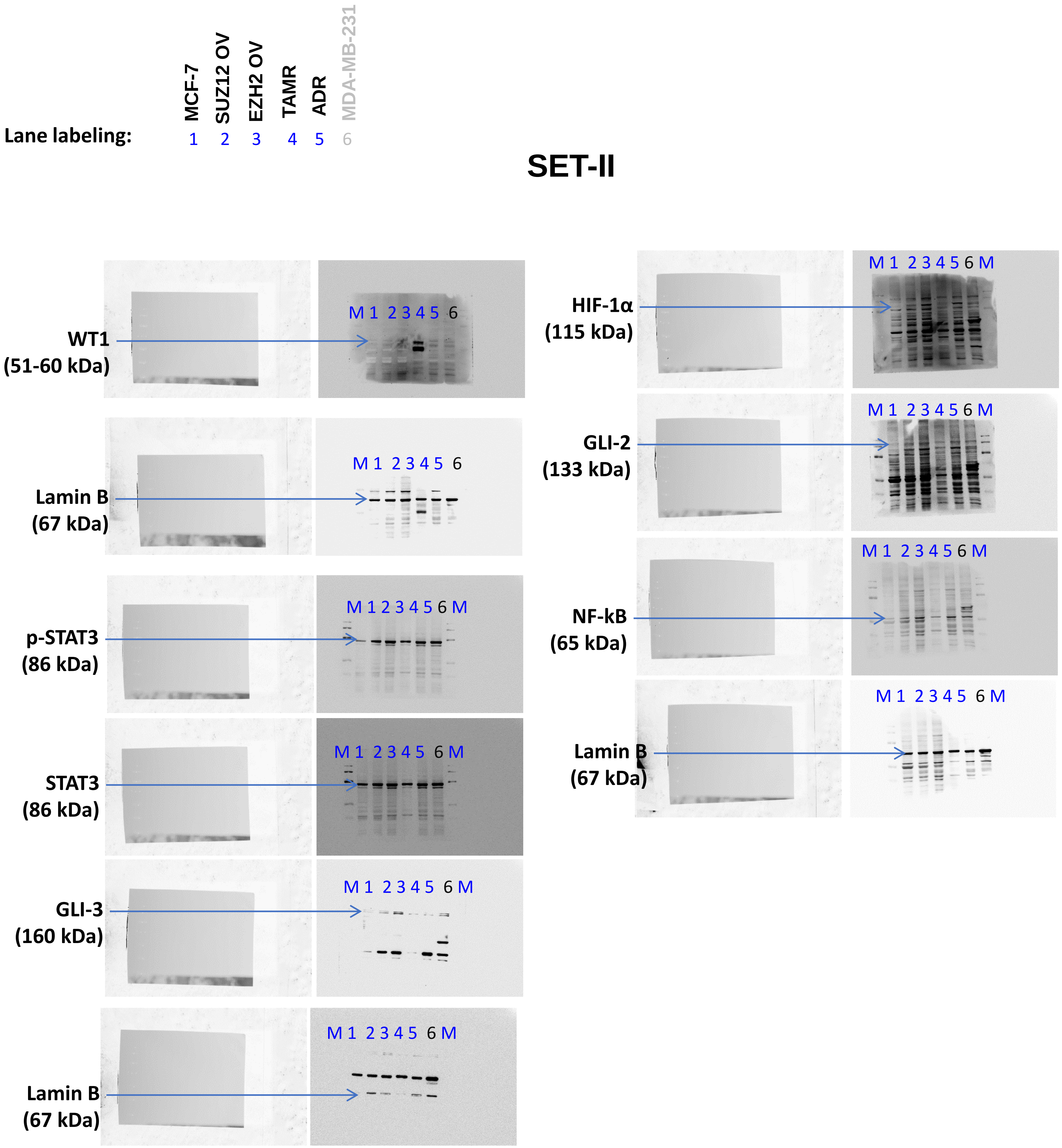

Lane labeling:
MCF-7 1
SUZ12 OV 2
EZH2 OV 3
TAMR 4
ADR 5
MDA-MB-231 6
SET-II
WT1 (51-60 kDa)
Lamin B (67 kDa)
p-STAT3 (86 kDa)
STAT3 (86 kDa)
GLI-3 (160 kDa)
Lamin B (67 kDa)
HIF-1α (115 kDa)
GLI-2 (133 kDa)
NF-kB (65 kDa)
Lamin B (67 kDa)
M 1 2 3 4 5 6 M

Fig. 3B

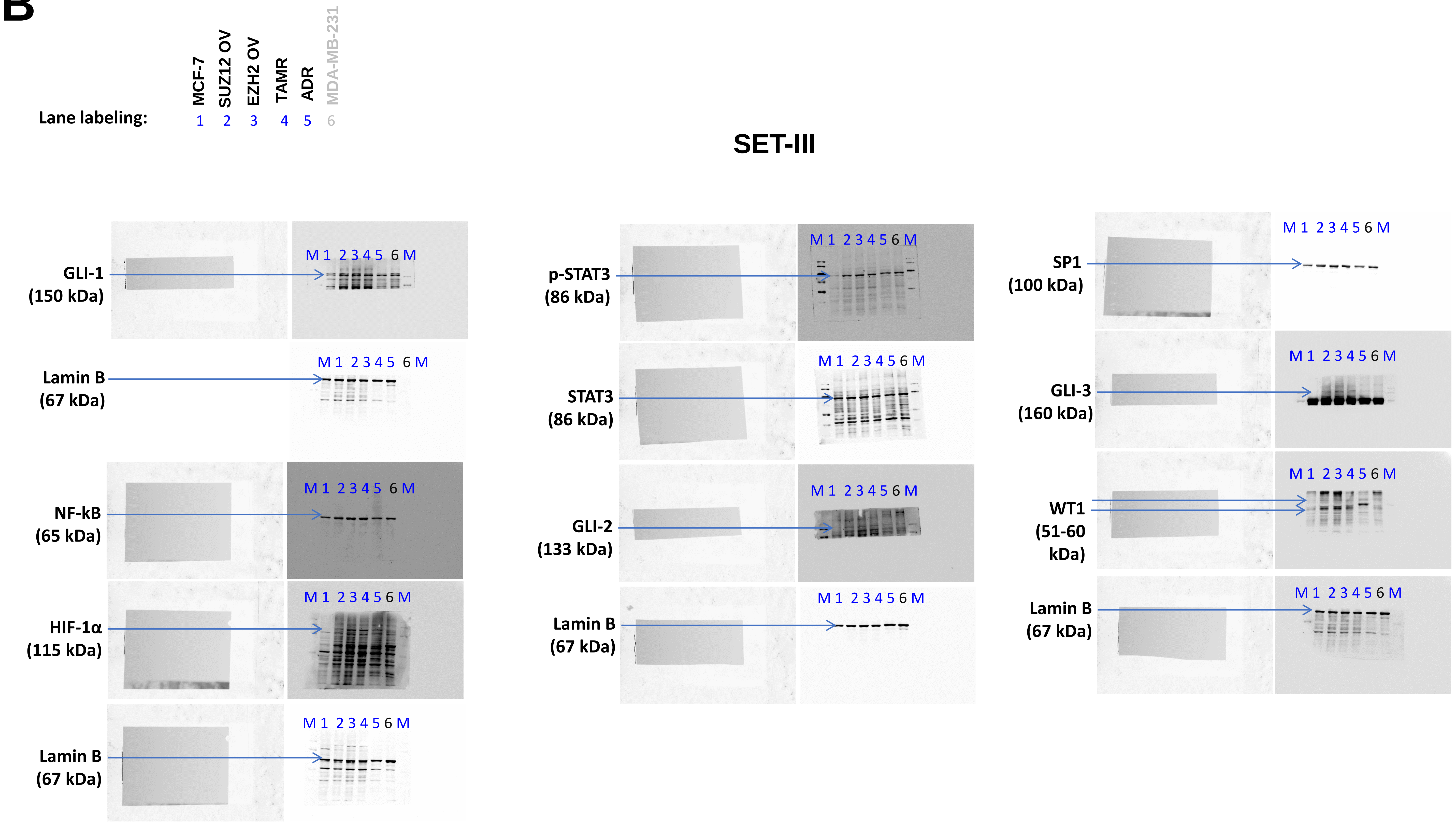

Lane labeling:
MCF-7
SUZ12 OV
EZH2 OV
TAMR
ADR
MDA-MB-231
1 2 3 4 5 6
SET-III
GLI-1 (150 kDa)
Lamin B (67 kDa)
NF-kB (65 kDa)
HIF-1α (115 kDa)
Lamin B (67 kDa)
p-STAT3 (86 kDa)
STAT3 (86 kDa)
GLI-2 (133 kDa)
Lamin B (67 kDa)
SP1 (100 kDa)
GLI-3 (160 kDa)
WT1 (51-60 kDa)
Lamin B (67 kDa)
M 1 2 3 4 5 6 M

Fig. 3C

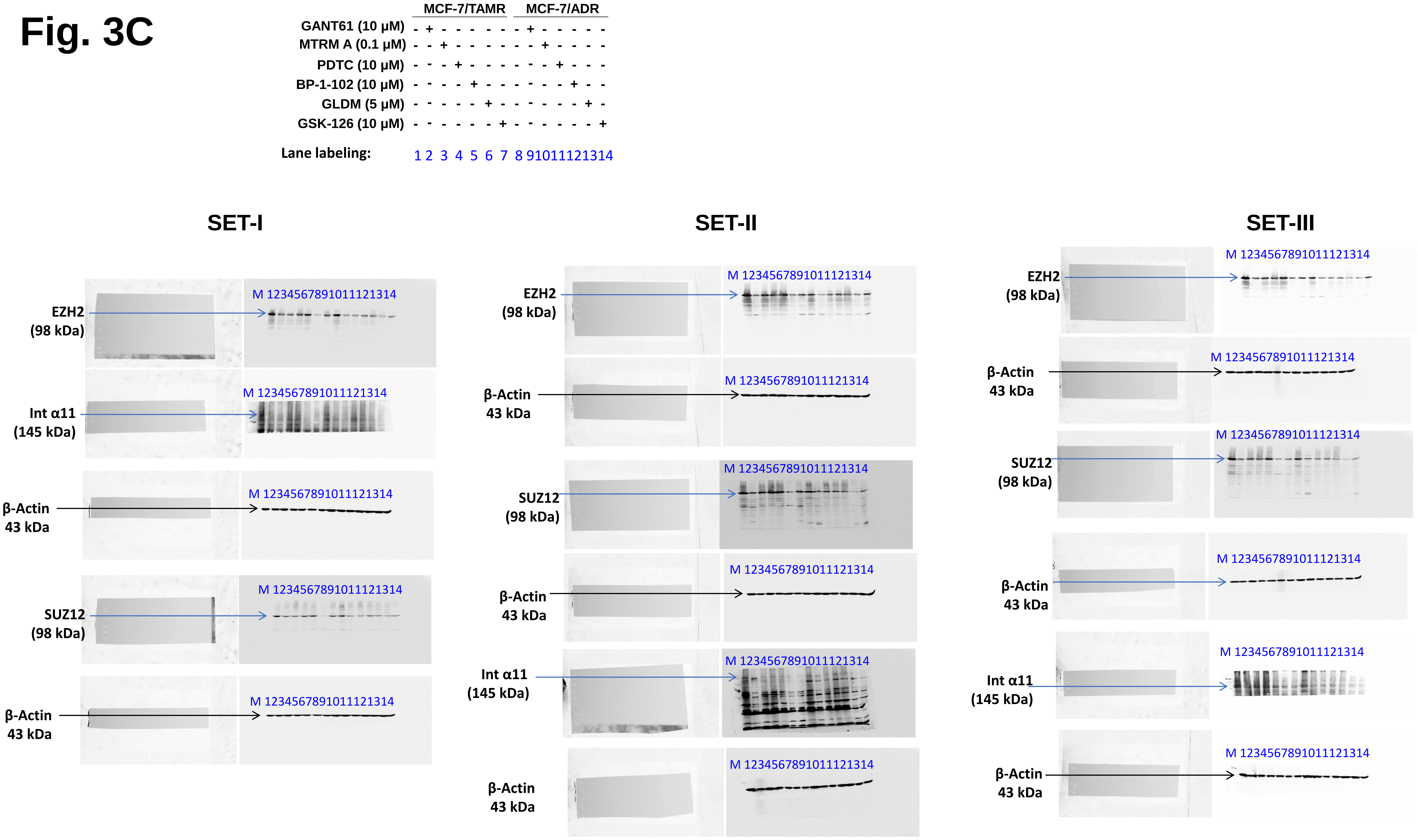
MCF-7/TAMR
MCF-7/ADR
GANT61 (10 µM) - + - - - - - - + - - - - -
MTRM A (0.1 µM) - - + - - - - - - + - - - -
PDTC (10 µM) - - - + - - - - - - + - - -
BP-1-102 (10 µM) - - - - + - - - - - - + - -
GLDM (5 µM) - - - - - + - - - - - - + -
GSK-126 (10 µM) - - - - - - + - - - - - - +
Lane labeling:
1 2 3 4 5 6 7 8 91011121314
SET-I
SET-II
SET-III
EZH2
(98 kDa)
Int α11
(145 kDa)
β-Actin
43 kDa
SUZ12
(98 kDa)
M 1234567891011121314

Fig. 3D

	TAMR				ADR			
siNT	+	-	-	-	+	-	-	-
siHIF-1α	-	+	-	-	-	+	-	-
siGLI-1	-	-	+	-	-	-	+	-
siEZH2	-	-	-	+	-	-	-	+
Lane labeling:	1	2	3	4	5	6	7	8

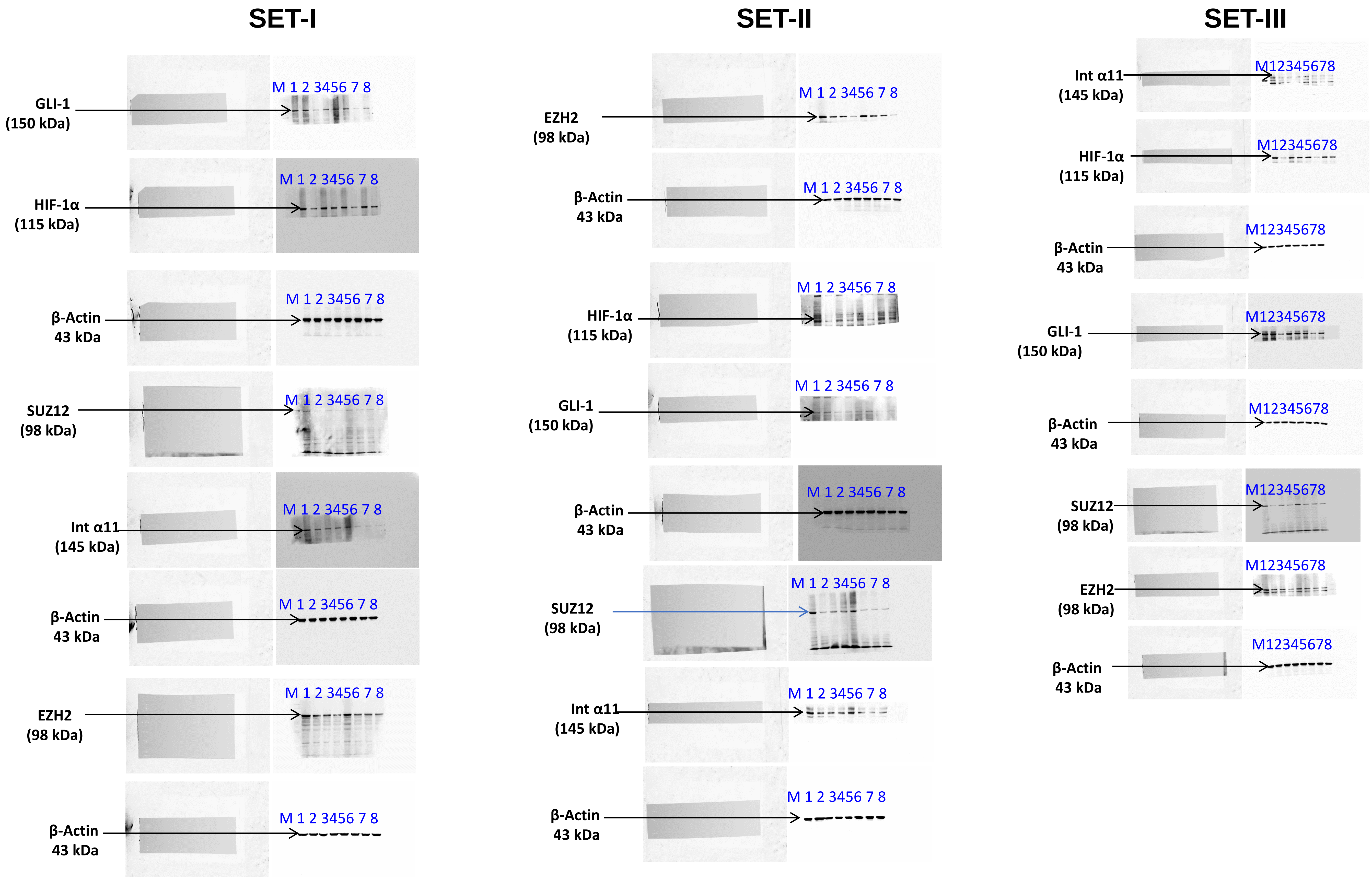

Fig. 4A

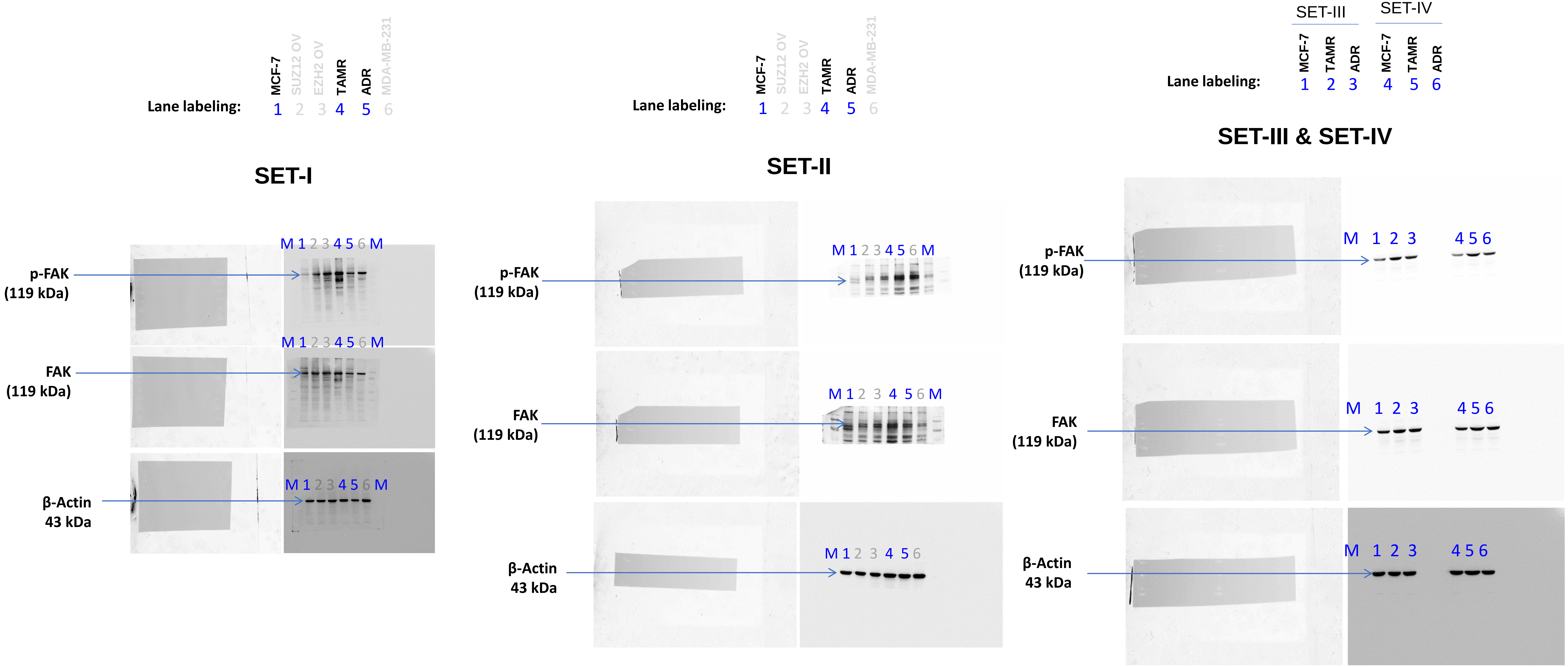
Lane labeling:
MCF-7
SUZ12 OV
EZH2 OV
TAMR
ADR
MDA-MB-231
1 2 3 4 5 6
SET-I
M 1 2 3 4 5 6 M
p-FAK
(119 kDa)
FAK
(119 kDa)
β-Actin
43 kDa
SET-II
SET-III
SET-IV
SET-III & SET-IV
M 1 2 3 4 5 6

Fig. 4B

	TAMR				ADR			
siNT	+	-	-	-	+	-	-	-
siITGA11	-	+	-	-	-	+	-	-
siSUZ12	-	-	+	-	-	-	+	-
siEZH2	-	-	-	+	-	-	-	+
Lane labeling:	1	2	3	4	5	6	7	8

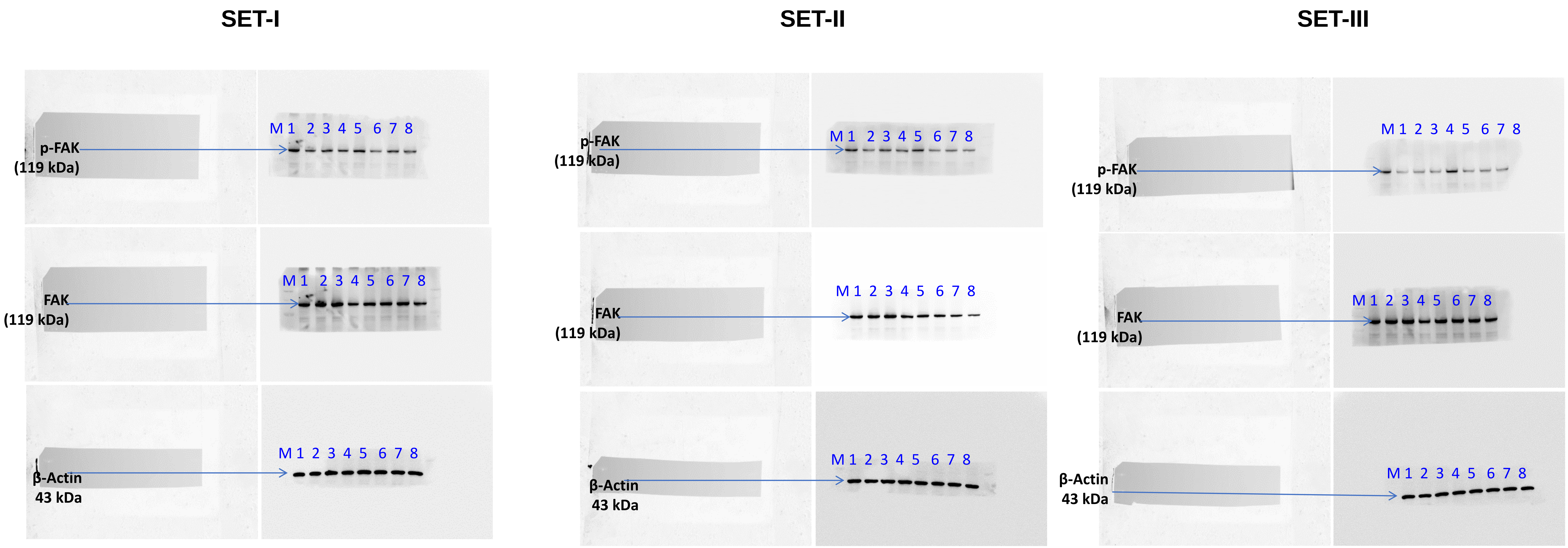

Fig. 4C

SET-I

	TAMR				ADR			
Ifebemtinib (µM)	-	1	5	10	-	1	5	10
Lane labeling:	1	2	3	4	5	6	7	8

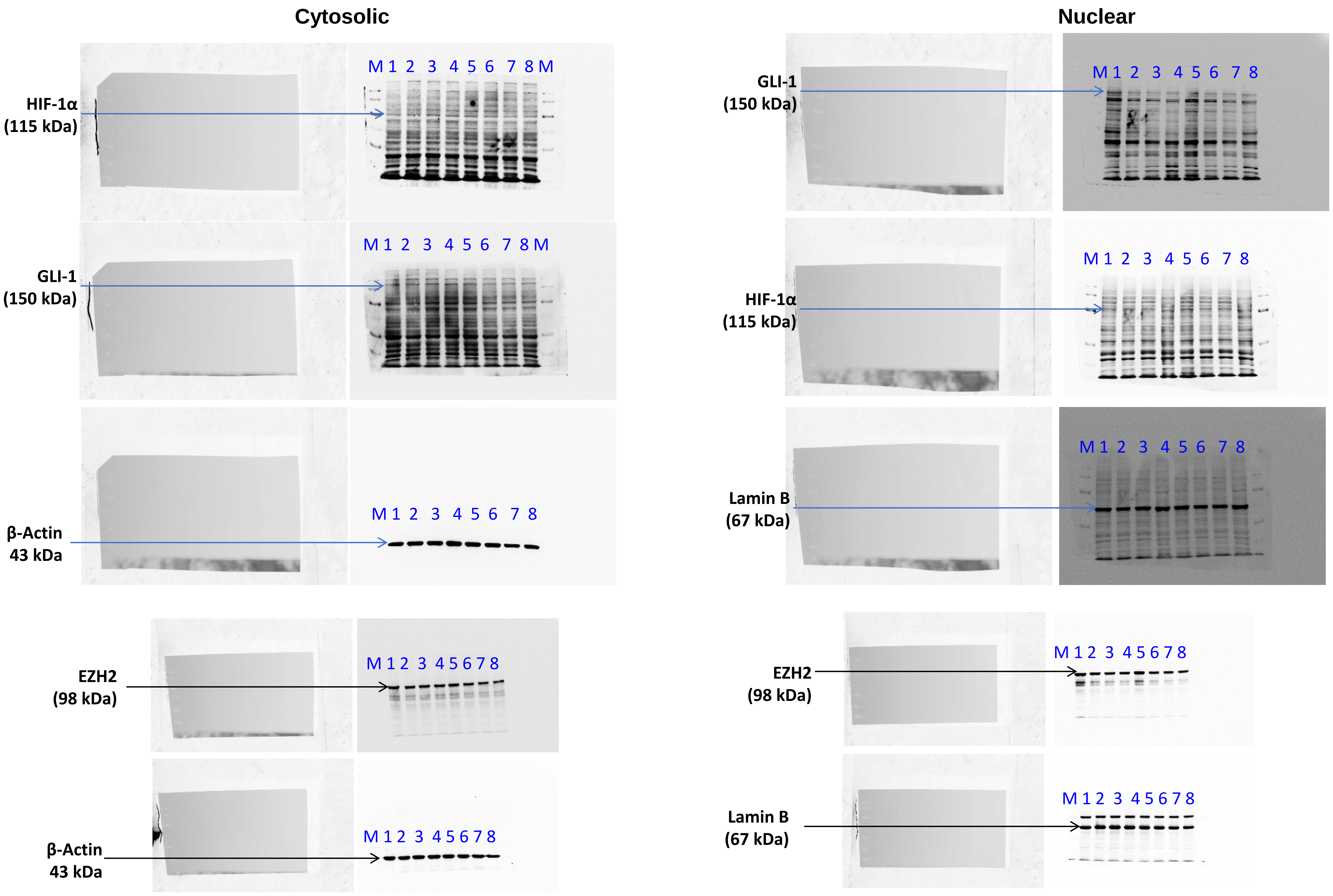

Fig. 4C

SET-II

	TAMR				ADR			
Ifebemtinib (μM)	-	1	5	10	-	1	5	10
Lane labeling:	1	2	3	4	5	6	7	8

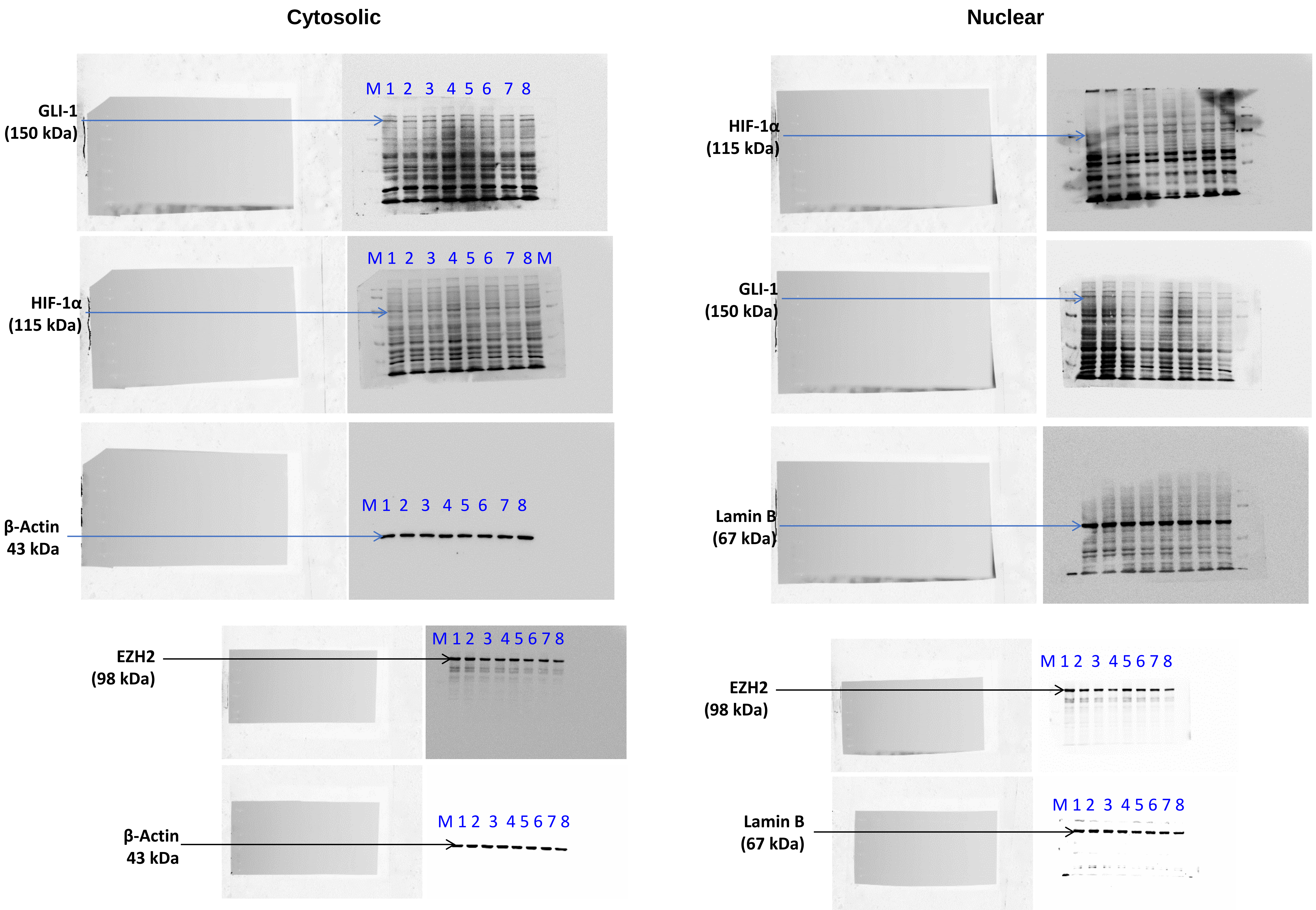

Fig. 4C

SET-III

	TAMR				ADR			
Ifebemtinib (μM)	-	1	5	10	-	1	5	10
Lane labeling:	1	2	3	4	5	6	7	8

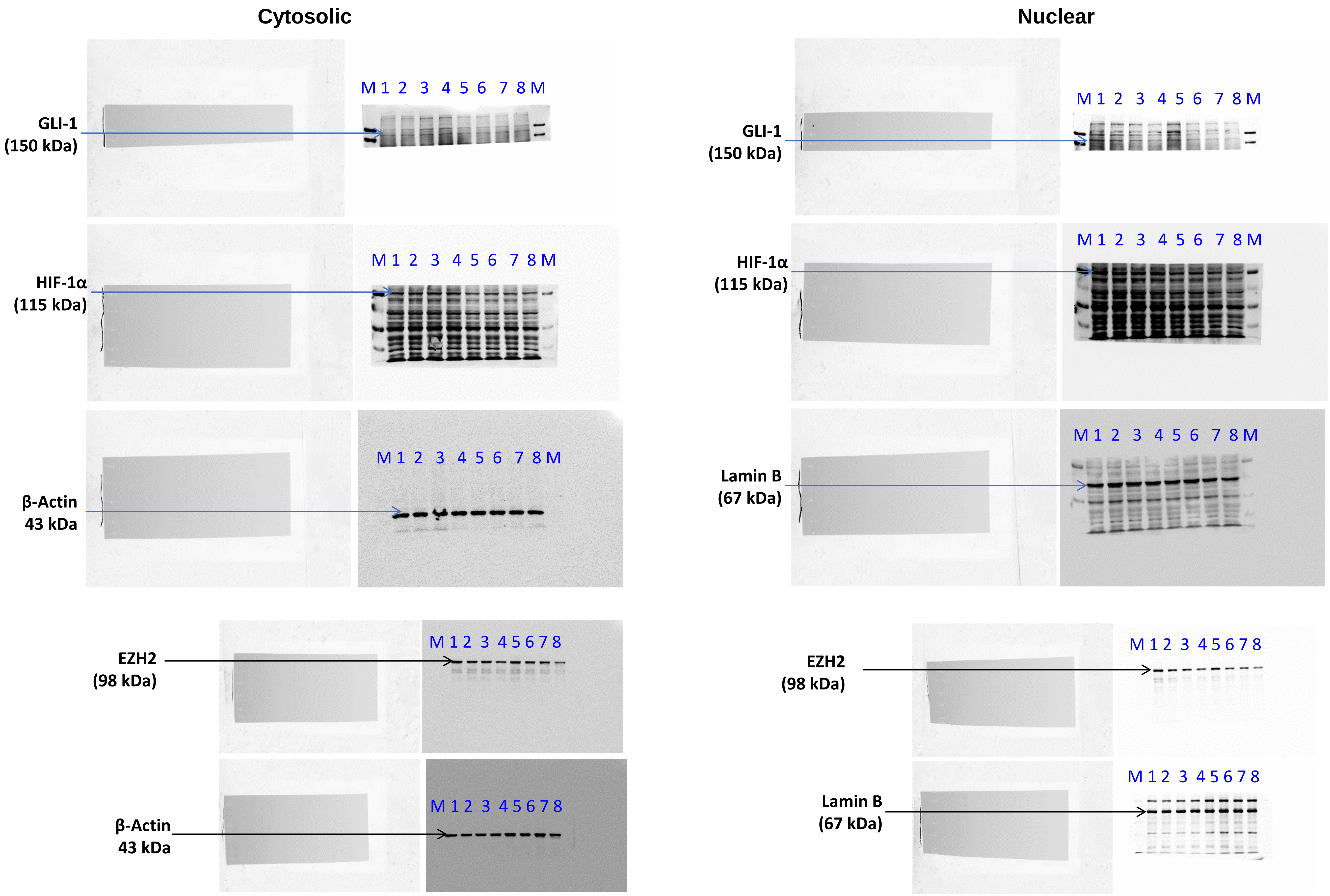

Fig. 4D

	TAMR			ADR		
siNT	-	+	-	-	+	-
siITGA11	-	-	+	-	-	+
**Lane labeling:**	1	2	3	4	5	6

SET-I

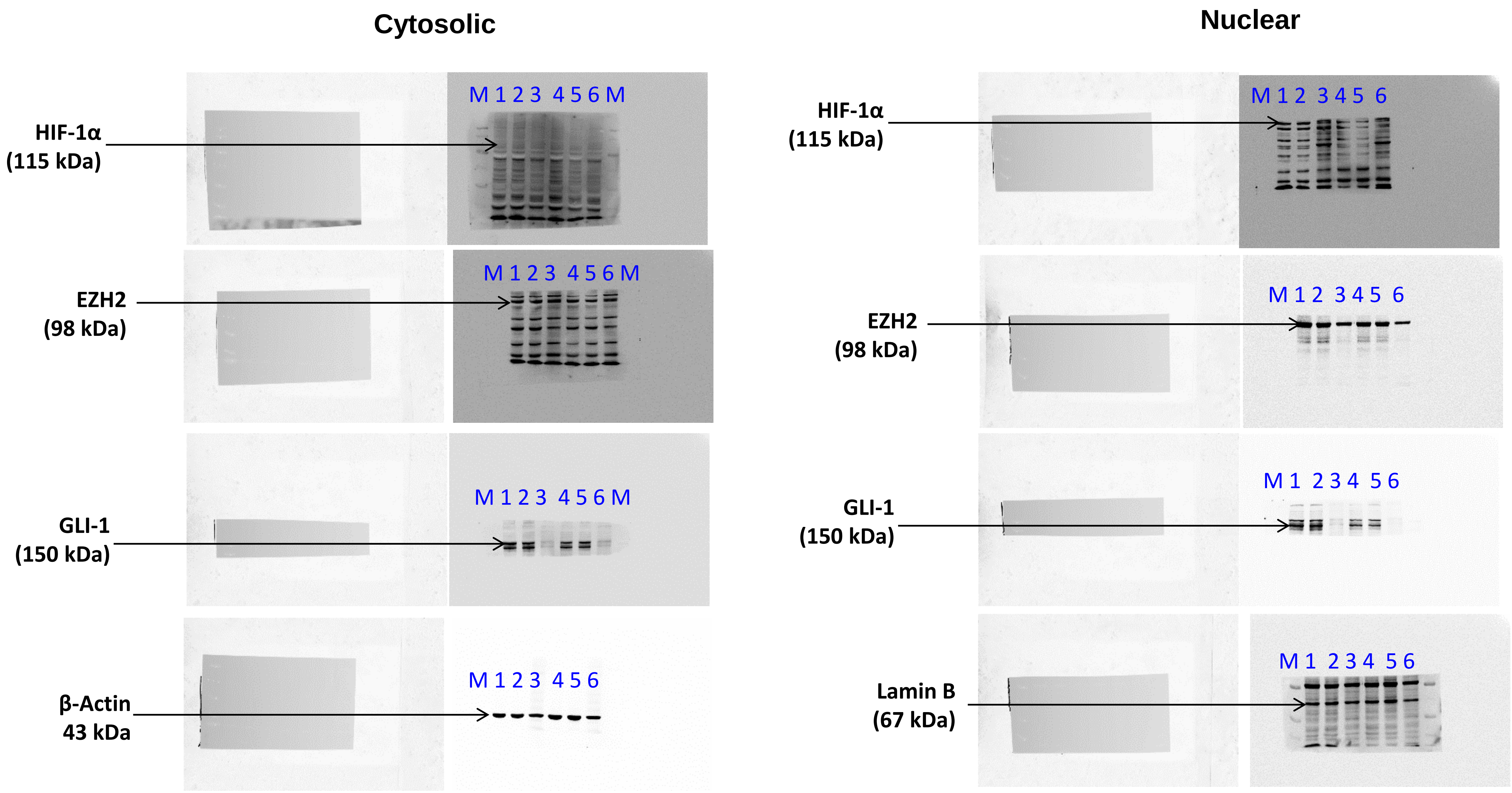

Fig. 4D

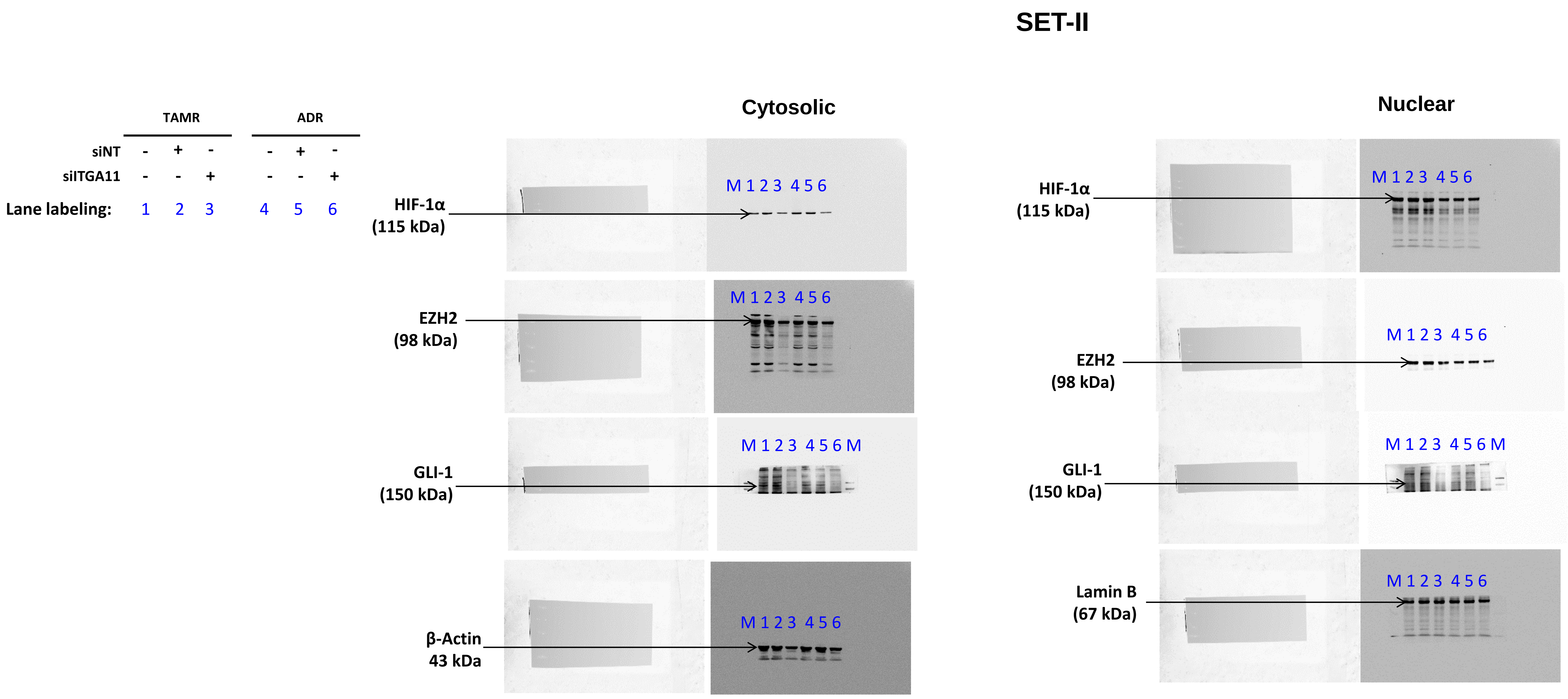
SET-II
TAMR
ADR
siNT - + - - + -
siITGA11 - - + - - +
Lane labeling: 1 2 3 4 5 6
Cytosolic
Nuclear
HIF-1α
(115 kDa)
M 1 2 3 4 5 6
EZH2
(98 kDa)
M 1 2 3 4 5 6
GLI-1
(150 kDa)
M 1 2 3 4 5 6 M
β-Actin
43 kDa
Lamin B
(67 kDa)

Fig. 4D

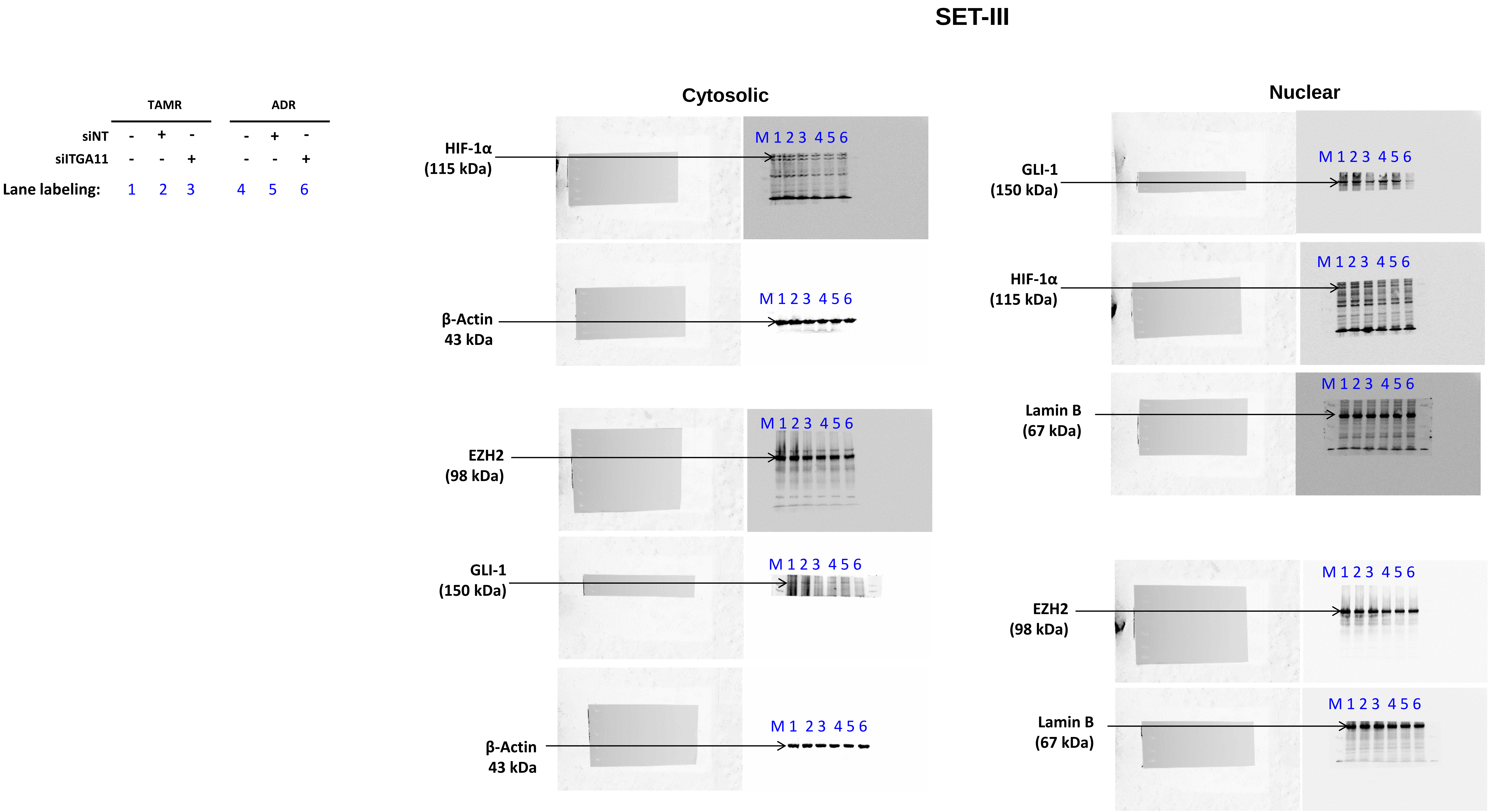
SET-III
TAMR
ADR
siNT - + - - + -
siITGA11 - - + - - +
Lane labeling: 1 2 3 4 5 6
Cytosolic
HIF-1α (115 kDa)
β-Actin 43 kDa
EZH2 (98 kDa)
GLI-1 (150 kDa)
β-Actin 43 kDa
Nuclear
GLI-1 (150 kDa)
HIF-1α (115 kDa)
Lamin B (67 kDa)
EZH2 (98 kDa)
Lamin B (67 kDa)
M 1 2 3 4 5 6

Fig. 5C

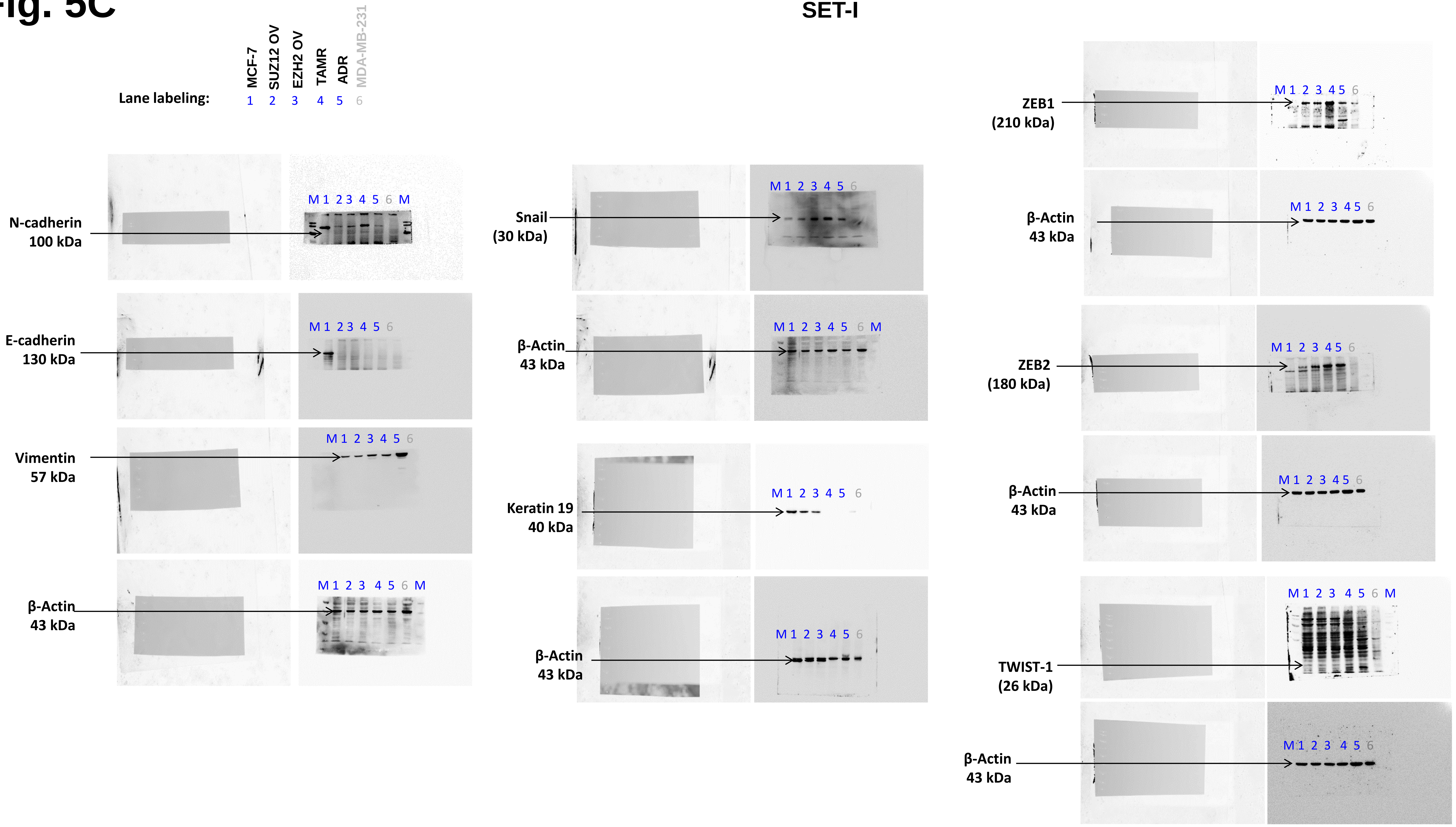
SET-I
Lane labeling:
MCF-7 1
SUZ12 OV 2
EZH2 OV 3
TAMR 4
ADR 5
MDA-MB-231 6
N-cadherin 100 kDa
E-cadherin 130 kDa
Vimentin 57 kDa
β-Actin 43 kDa
Snail (30 kDa)
β-Actin 43 kDa
Keratin 19 40 kDa
β-Actin 43 kDa
ZEB1 (210 kDa)
β-Actin 43 kDa
ZEB2 (180 kDa)
β-Actin 43 kDa
TWIST-1 (26 kDa)
β-Actin 43 kDa
M 1 2 3 4 5 6 M

Fig. 5C

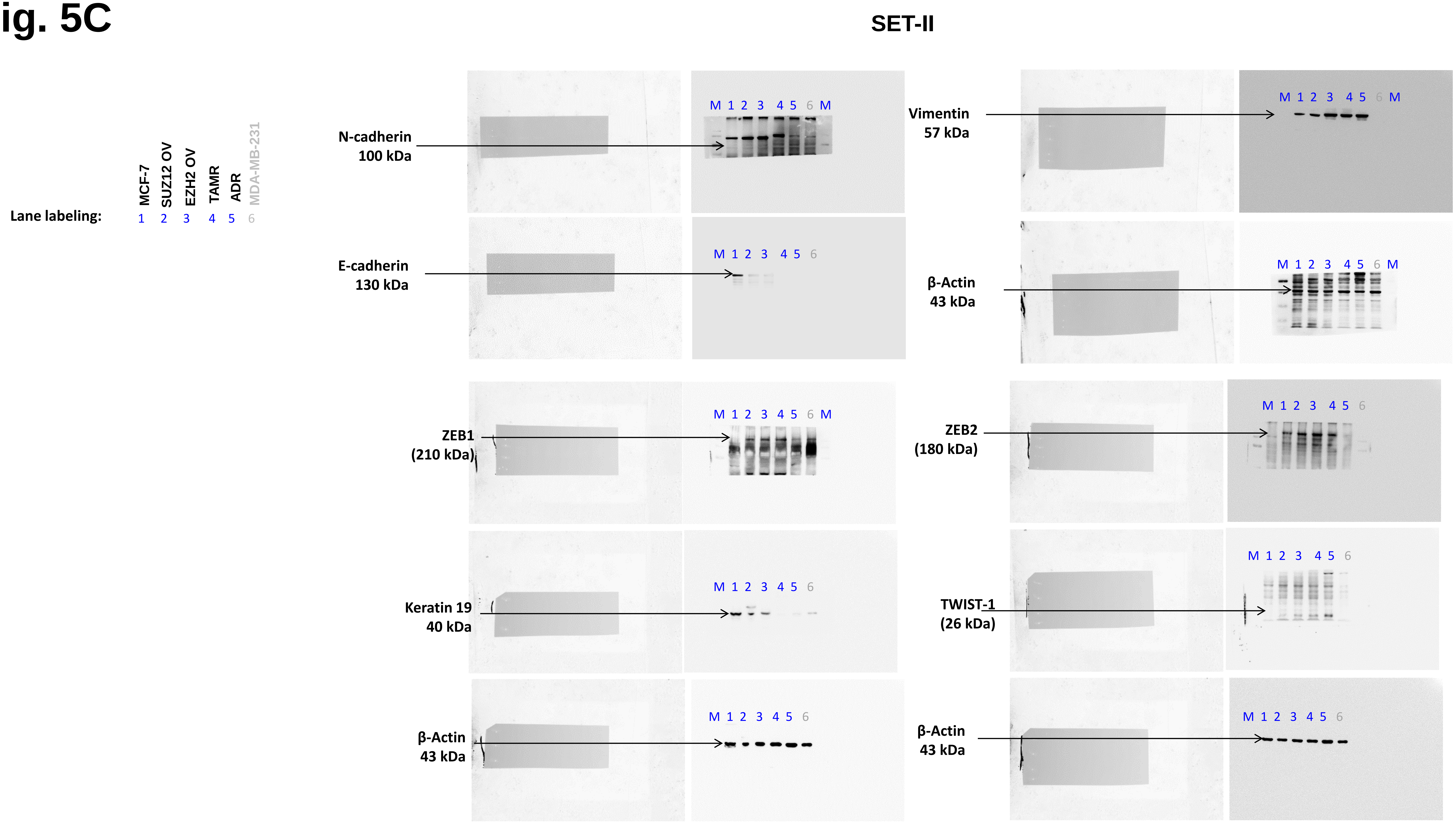
SET-II
Lane labeling:
1 MCF-7
2 SUZ12 OV
3 EZH2 OV
4 TAMR
5 ADR
6 MDA-MB-231
N-cadherin
100 kDa
M 1 2 3 4 5 6 M
Vimentin
57 kDa
M 1 2 3 4 5 6 M
E-cadherin
130 kDa
M 1 2 3 4 5 6
β-Actin
43 kDa
M 1 2 3 4 5 6 M
ZEB1
(210 kDa)
M 1 2 3 4 5 6 M
ZEB2
(180 kDa)
M 1 2 3 4 5 6
Keratin 19
40 kDa
M 1 2 3 4 5 6
TWIST-1
(26 kDa)
M 1 2 3 4 5 6
β-Actin
43 kDa
M 1 2 3 4 5 6
β-Actin
43 kDa
M 1 2 3 4 5 6

Fig. 5C

SET-III

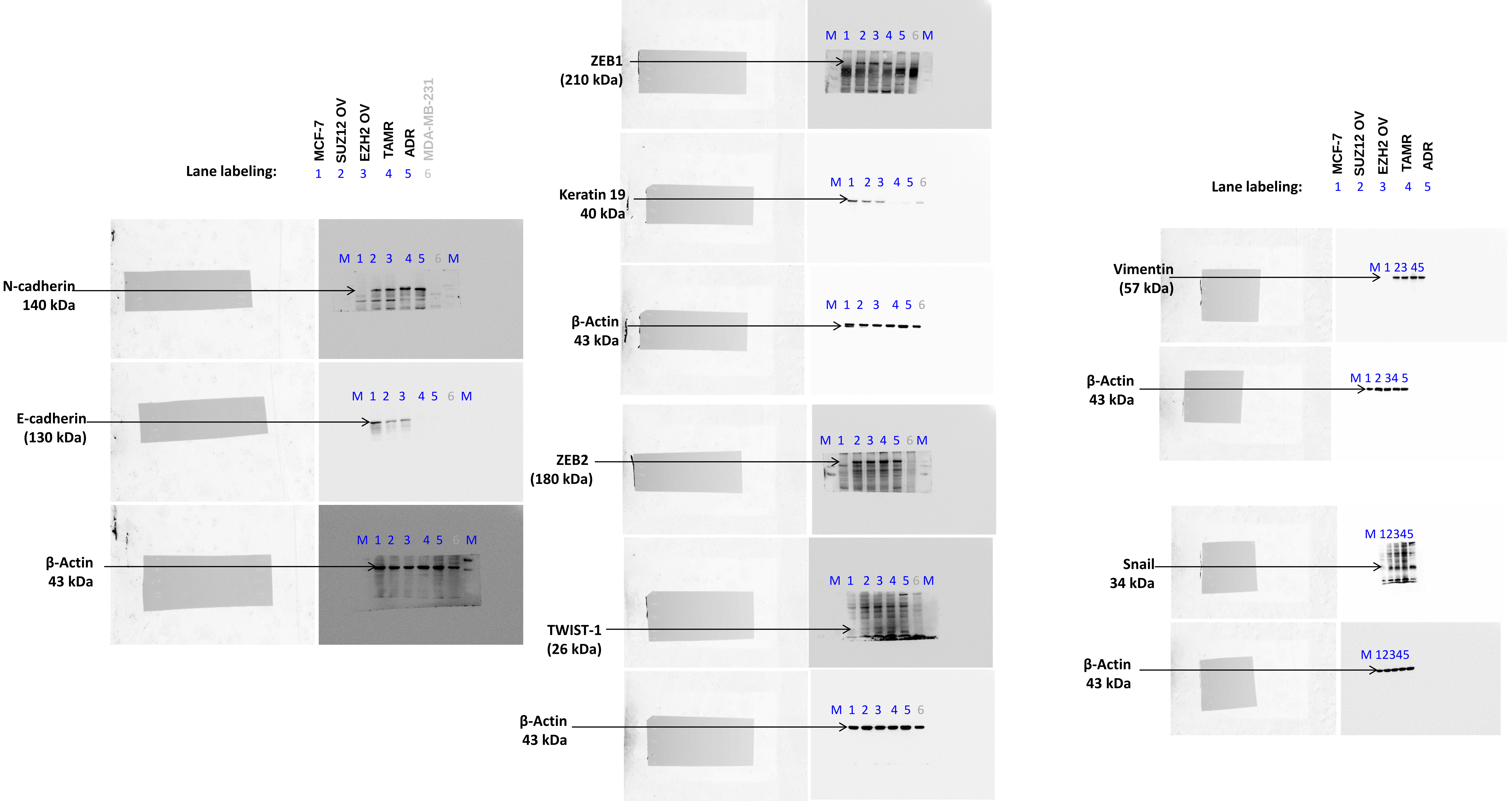

Fig. 5D

SET-I

	MCF-7/ADR				MCF-7/TAMR			
siNT	+	-	-	-	+	-	-	-
siITGA11	-	+	-	-	-	+	-	-
siSUZ12	-	-	+	-	-	-	+	-
siEZH2	-	-	-	+	-	-	-	+
**Lane labeling:**	1	2	3	4	5	6	7	8

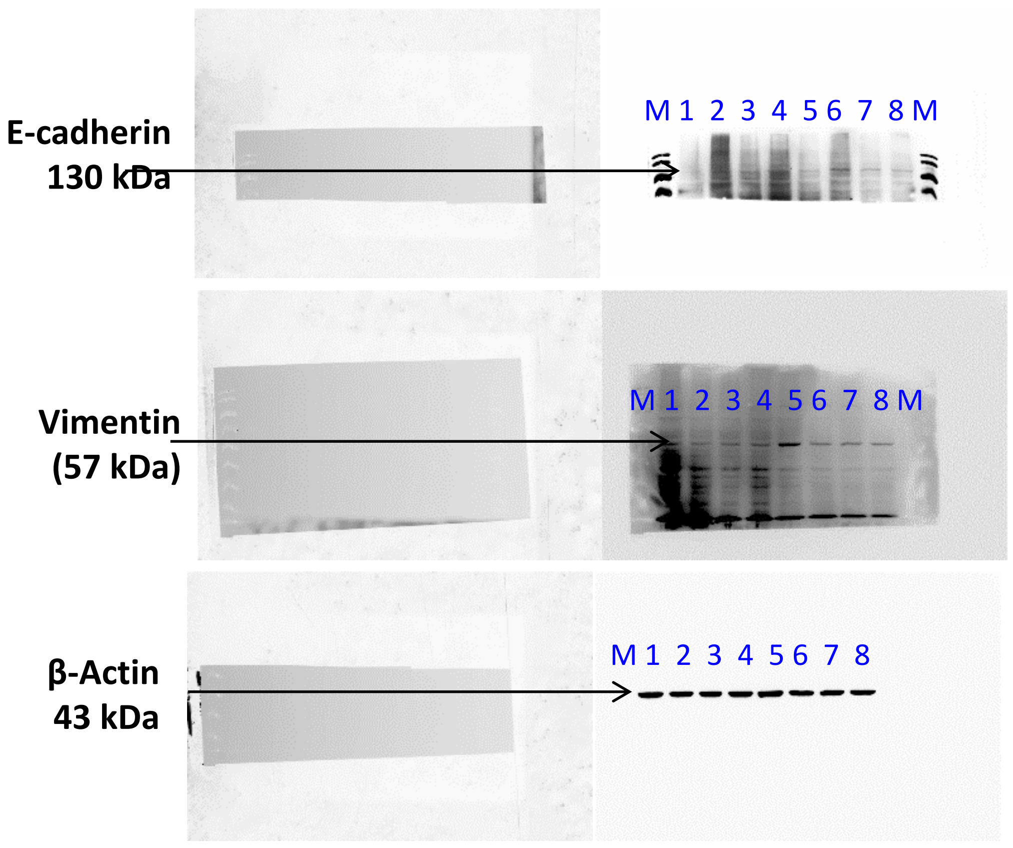

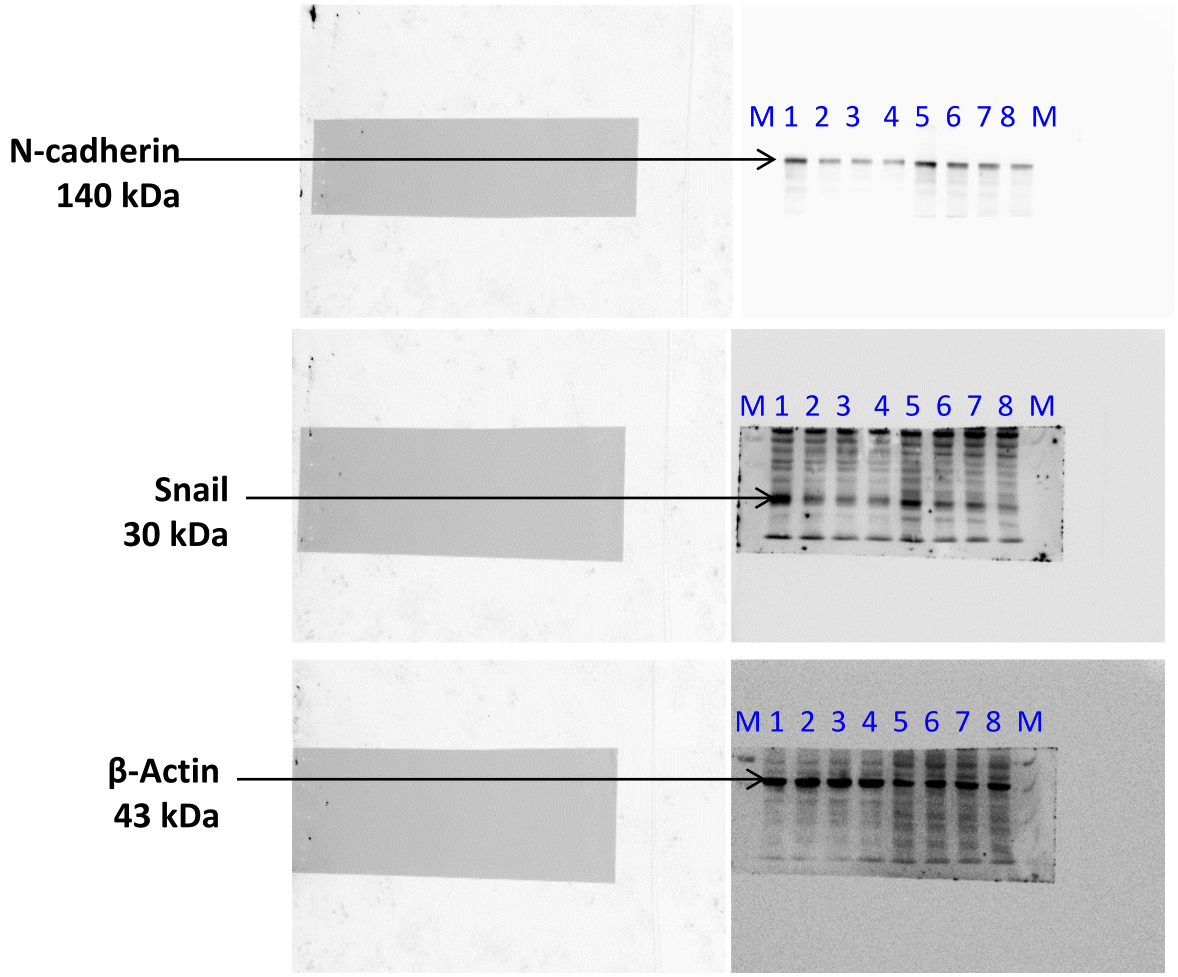

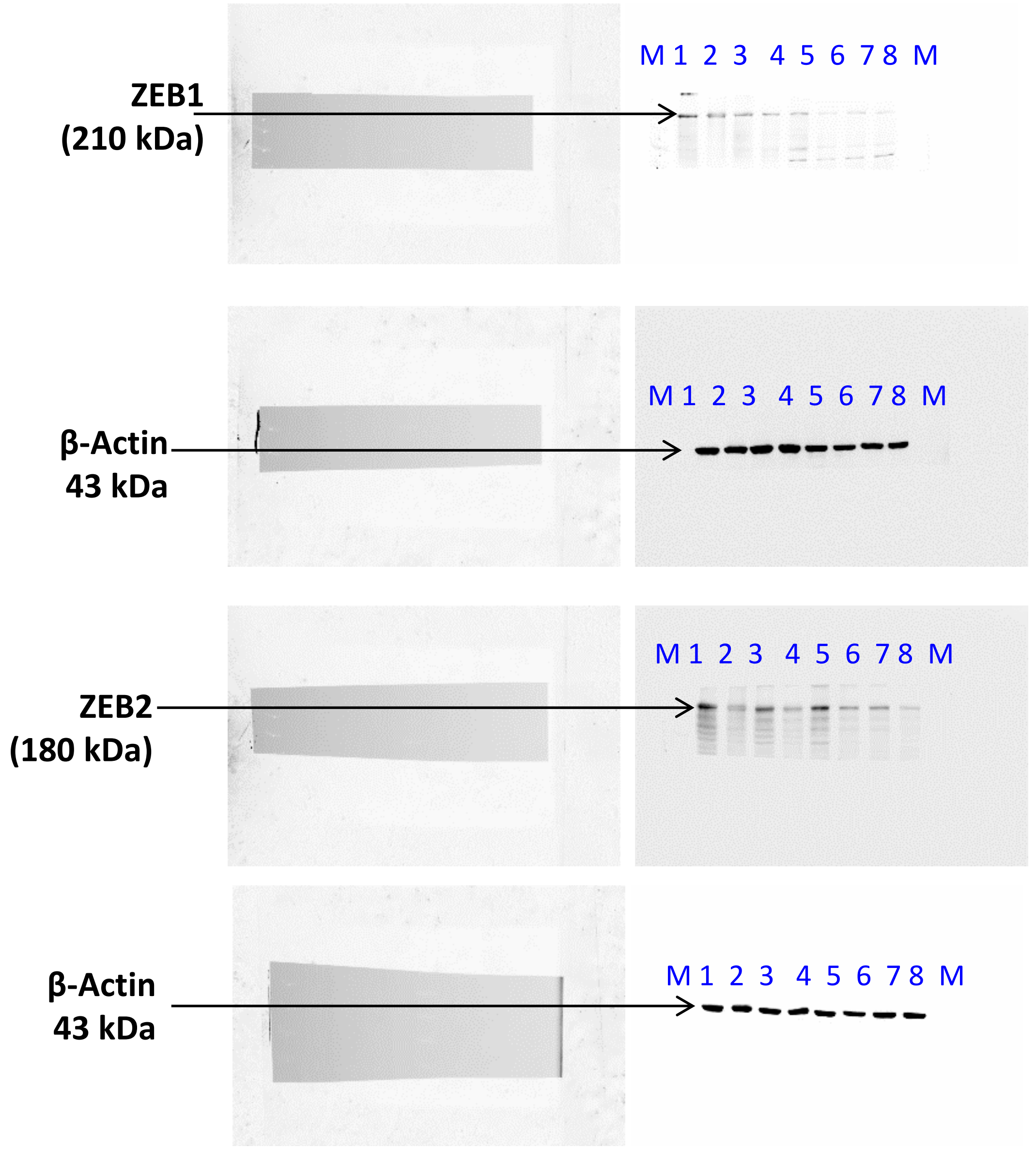

Fig. 5D

SET-II

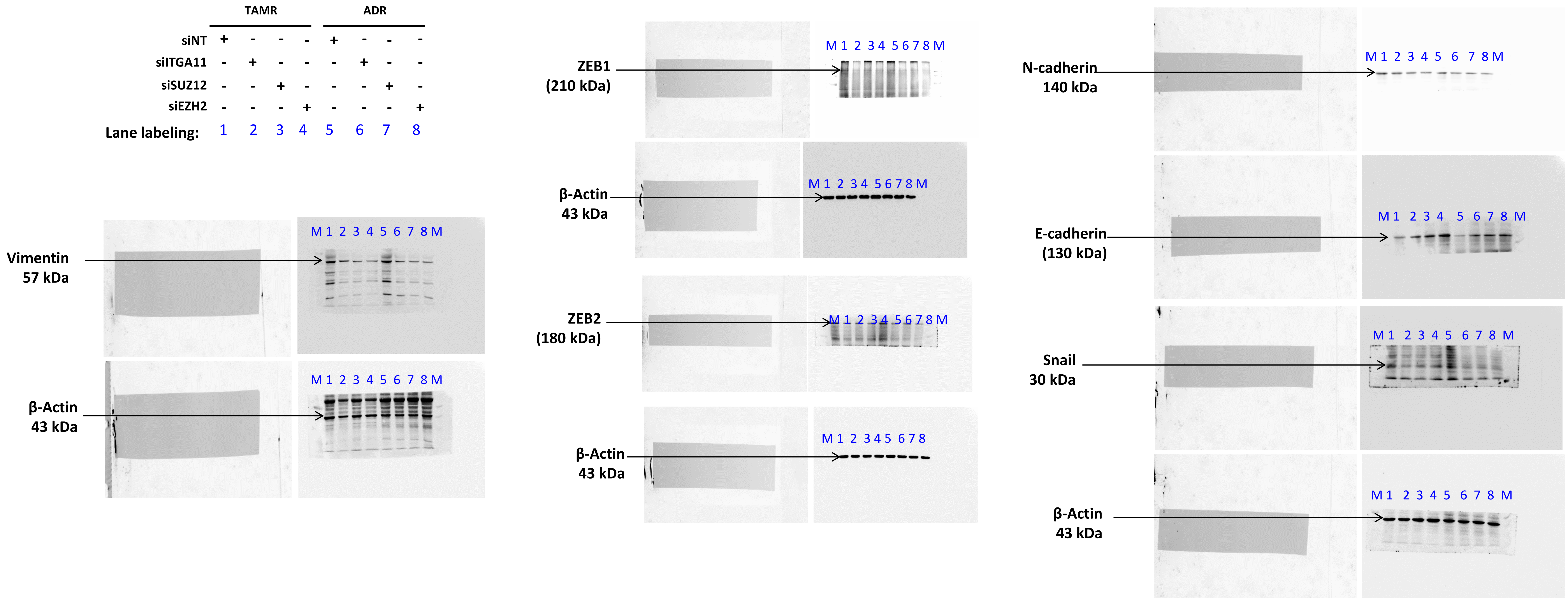
TAMR
ADR
siNT + - - - + - - -
siITGA11 - + - - - + - -
siSUZ12 - - + - - - + -
siEZH2 - - - + - - - +
Lane labeling: 1 2 3 4 5 6 7 8
Vimentin
57 kDa
M 1 2 3 4 5 6 7 8 M
β-Actin
43 kDa
M 1 2 3 4 5 6 7 8 M
ZEB1
(210 kDa)
M 1 2 3 4 5 6 7 8 M
β-Actin
43 kDa
M 1 2 3 4 5 6 7 8 M
ZEB2
(180 kDa)
M 1 2 3 4 5 6 7 8 M
β-Actin
43 kDa
M 1 2 3 4 5 6 7 8
N-cadherin
140 kDa
M 1 2 3 4 5 6 7 8 M
E-cadherin
(130 kDa)
M 1 2 3 4 5 6 7 8 M
Snail
30 kDa
M 1 2 3 4 5 6 7 8 M
β-Actin
43 kDa
M 1 2 3 4 5 6 7 8 M

Fig. 5D

SET-III

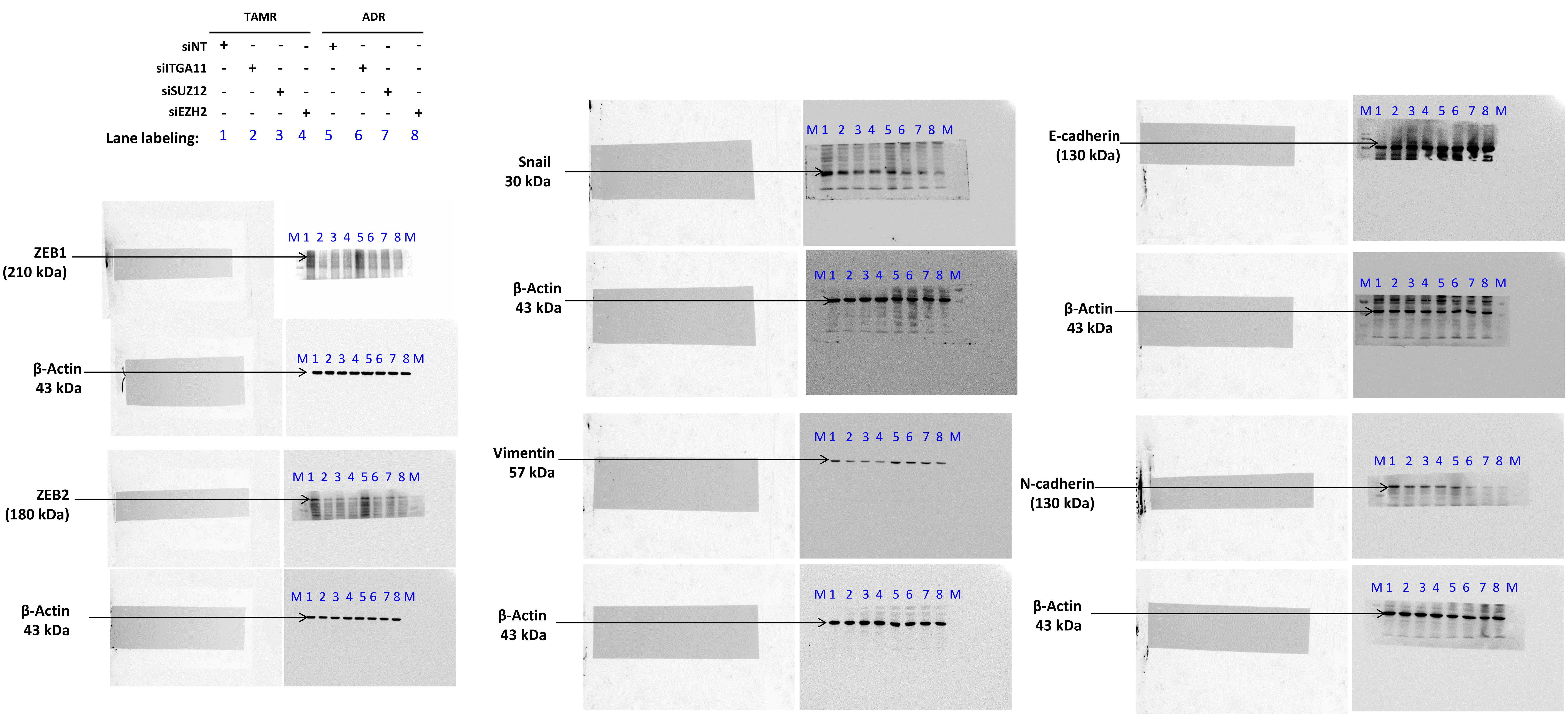

Supplement: Supplementary file 2 — Supplementary Material 2 [file 13058_2024_1827_MOESM2_ESM.pdf]
